# Cardiovascular Outcomes and the Physical and Chemical Properties of Metal Ions Found in Particulate Matter Air Pollution: A QICAR Study

**DOI:** 10.1289/ehp.1205793

**Published:** 2013-03-05

**Authors:** Qingyu Meng, Jennifer Richmond-Bryant, Shou-En Lu, Barbara Buckley, William J. Welsh, Eric A. Whitsel, Adel Hanna, Karin B. Yeatts, Joshua Warren, Amy H. Herring, Aijun Xiu

**Affiliations:** 1 School of Public Health, University of Medicine and Dentistry of New Jersey, Piscataway, New Jersey, USA; 2 National Center for Environmental Assessment, U.S. Environmental Protection Agency, Research Triangle Park, North Carolina, USA; 3 Robert Wood Johnson Medical School, University of Medicine and Dentistry of New Jersey, Piscataway, New Jersey, USA; 4 Department of Medicine,; 5 Department of Epidemiology,; 6 Institute for the Environment, and; 7 Department of Biostatistics, University of North Carolina at Chapel Hill, Chapel Hill, North Carolina, USA

**Keywords:** air pollution, cardiovascular disease, multipollutant, QICAR, QSAR

## Abstract

Background: This paper presents an application of quantitative ion character–activity relationships (QICAR) to estimate associations of human cardiovascular (CV) diseases (CVDs) with a set of metal ion properties commonly observed in ambient air pollutants. QICAR has previously been used to predict ecotoxicity of inorganic metal ions based on ion properties.

Objectives: The objective of this work was to examine potential associations of biological end points with a set of physical and chemical properties describing inorganic metal ions present in exposures using QICAR.

Methods: Chemical and physical properties of 17 metal ions were obtained from peer-reviewed publications. Associations of cardiac arrhythmia, myocardial ischemia, myocardial infarction, stroke, and thrombosis with exposures to metal ions (measured as inference scores) were obtained from the Comparative Toxicogenomics Database (CTD). Robust regressions were applied to estimate the associations of CVDs with ion properties.

Results: CVD was statistically significantly associated (Bonferroni-adjusted significance level of 0.003) with many ion properties reflecting ion size, solubility, oxidation potential, and abilities to form covalent and ionic bonds. The properties are relevant for reactive oxygen species (ROS) generation, which has been identified as a possible mechanism leading to CVDs.

Conclusion: QICAR has the potential to complement existing epidemiologic methods for estimating associations between CVDs and air pollutant exposures by providing clues about the underlying mechanisms that may explain these associations.

Changes in the cardiovascular (CV) system may occur if particulate matter (PM) exposure initiates pulmonary oxidative stress and inflammation and/or pulmonary reflex responses. These responses can lead to adverse outcomes such as stroke, myocardial ischemia, and myocardial infarction [Brook et al. 2004, 2010; Chuang et al. 2007; Mills et al. 2007; U.S. Environmental Protection Agency (EPA) 2009; Zhang et al. 2009], as illustrated by the potential pathways for PM to affect the CV system (Figure 1). Oxidative stress occurs when the burden of reactive oxygen species [ROS (oxygen radicals and non-radical oxygen derivatives)] at a target site is larger than the target site’s antioxidant reserve (Halliwell and Gutteridge 2007). Oxidative stress can lead to altered cell signaling, DNA injury, or apoptosis (U.S. EPA 2009). In PM experimental exposure studies of *in vitro* macrophage cytotoxicity (Becker et al. 2002; Hatch et al. 1985) and *in vivo* intratracheal instillation in mice, oxidative stress was associated more strongly with PM components such as metal ions than with PM mass. Oxidative stress induced by exposure to metal ions can occur directly or indirectly (Ercal et al. 2001). When occurring directly, oxidation–reduction (redox)-active metal ions in PM, such as iron (Fe) and copper (Cu), have been shown to cause ROS formation through experimental *in vitro* testing of normal human bronchial epithelial and alveolar macrophage cells (Becker et al. 2005), an electron paramagnetic resonance assay (Boogaard et al. 2012), and a dithiothreitol assay (Cho et al. 2005) to replicate oxidation in the lung. Redox-active metal ions in PM participate in the Fenton reaction to produce the hydroxyl radical (OH^•^), which is subsequently involved in ROS production (Cho et al. 2005; Shafer et al. 2010). Redox-inactive metal ions, such as cadmium (Cd) and lead (Pb), can reduce antioxidant levels in cells by forming complexes with antioxidants; this condition leaves the cell vulnerable to oxidation, as described by Ercal et al. (2001) and observed through imaging experiments employing hydrogen peroxide–specific and redox-specific fluorophores (Cheng et al. 2010). PM components, including metal ions, have been shown to move into the circulation in animal models (Nemmar et al. 2001; Oberdörster et al. 2002) and controlled human exposure studies (Nemmar et al. 2002), but it is unclear whether they remain free or become sequestered (Brook et al. 2004). The location of oxidative stress within the body can also be affected by other ion properties, such as solubility in lipids or epithelial lining fluid and electron exchange properties of metal ions, for both redox-active and redox-inactive metal ions (Miyata and van Eeden 2011). A rat inhalation study suggested that exposure to metal ions in PM may cause oxidative stress in the lung, leading to the production and release of proinflammatory cytokines and endothelin-1 into the circulatory system (Thomson et al. 2005). These mediators could then travel to the heart and blood vessels, where they may mediate downstream inflammatory effects (Brook et al. 2010). Alternatively, an epidemiology panel study of greater Boston-area coronary artery disease patients exhibiting ST segment depression suggested that exposure to both gaseous and particulate air pollutants may lead to the activation of pulmonary reflexes and local inflammation and subsequent alteration of the autonomic nervous system, and resulting heart rate variability changes (Chuang et al. 2008). Markers of inflammation and autonomic imbalance have also been associated with exposure to gaseous and particulate air pollutants in an epidemiology panel of healthy young adults in Taipei, China (Chuang et al. 2007). We hypothesize that relevant physical and chemical properties of metal ions can be used to predict adverse CV outcomes initiated by oxidative stress.

**Figure 1:**
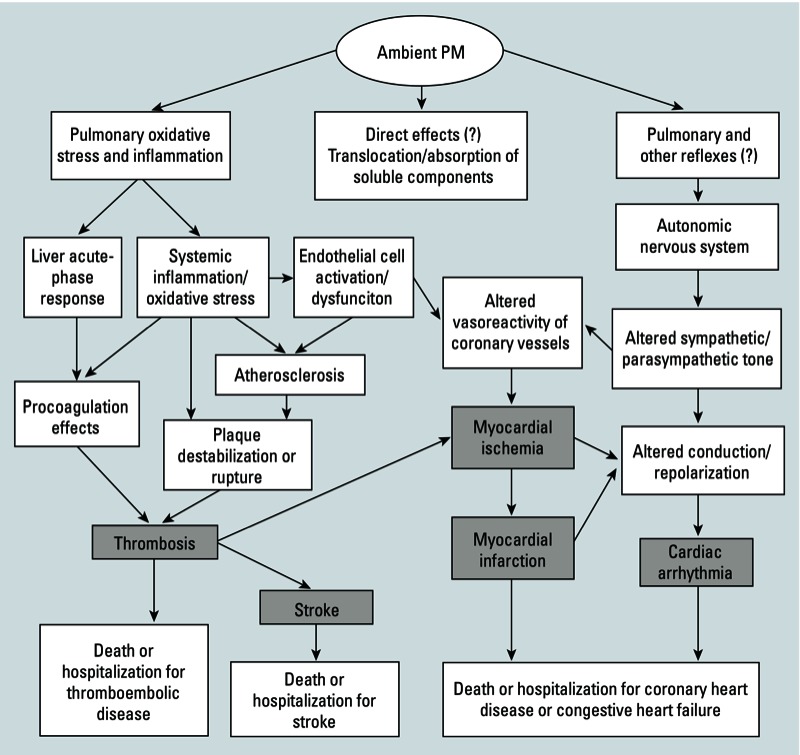
Potential pathways for effects of PM exposure on the cardiovascular system. The five end points examined here—arrhythmia, myocardial ischemia, myocardial infarction, thrombosis, and stroke—are shown on the diagram in gray boxes. Question marks denote areas of the potential mechanisms of action that are less certain. Conceptual model from Brook et al. (2010) and U.S. EPA (2009)

Quantitative ion character–activity relationships (QICARs) can be used to examine associations between biological end points and a set of physical and chemical properties describing inorganic metal ions present in exposures. Further, QICARs have been used to predict ecotoxicity of inorganic metal ions on the basis of a set of physical and chemical properties (e.g., McCloskey et al. 1996; Newman et al. 1998; Ownby and Newman 2003; Walker et al. 2003). For example, the lethal or effective concentrations for 50% of species have been predicted by the physical and chemical properties of metal ions such as softness (σ_p_) and the log of the first hydrolysis constant (|log(*K*_OH_)|) (Mendes et al. 2010; Ownby and Newman 2003; Williams et al. 1982). QICAR is a powerful tool for evaluating chemical toxicity because it allows for examination across compounds to determine which properties are more strongly associated with adverse responses. In turn, analysis of these properties may provide key insights into the biological mechanisms and pathways or target receptor(s) affected by metal ions. As mentioned above, health end points for humans [e.g. CV disease (CVD)] could also be linked with the physical and chemical properties of the chemicals to which people are exposed. However, to our knowledge, QICAR has not been applied in any human health studies.

The goal of this exploratory effort was to determine whether QICAR can be employed to study associations of adverse CVDs with human exposure to inorganic metal ions. QICAR appears to be a useful tool for elucidating important physical and chemical properties among redox-active and redox-inactive metal ions that may have adverse effects on CVD. In the present study, QICAR is applied to human health in the context of air pollution.

## Methods

*Data sources*. Chemical and physical property data were obtained from physical chemistry reference handbooks for 17 metal ions commonly found in the atmosphere: lithium [Li(I)], sodium [Na(I)], potassium [K(I)], cesium [Cs(I)], magnesium [Mg(II)], calcium [Ca(II)], barium [Ba(II)], manganese [Mn(II)], iron [Fe(II)], cobalt [Co(II)], nickel [Ni(II)], silver [Ag(I)], copper [Cu(II)], zinc [Zn(II)], cadmium [Cd(II)], mercury [Hg(II)], and lead [Pb(II)] (James and Lord 1992; Kaye and Laby 1993). These physicochemical property data have been applied in several peer-reviewed QICAR studies (McCloskey et al. 1996; Mendes et al. 2010; Newman et al. 1998; Ownby and Newman 2003; Walker et al. 2003; Williams et al. 1982). Several of these metal ions (Pb, Hg, Mn, Ni) are well-known toxicants (Dreher et al. 1997; Ercal et al. 2001; Lippmann et al. 2006; Moriwaki et al. 2008). Properties included in this QICAR examination (Table 1) are related to the exchange of electrons and solubility of metal ions and, therefore, may be associated with ROS generation (Mendes et al. 2010; Walker et al. 2003). These properties include fundamental attributes of metal ions (e.g., ion mass, ion length scale) as well as solubility, softness, tendency of an ion to be oxidized, energy required for oxidation, oxidation state, oxidation energy, ability to produce hydroxyl ions (OH^–^), and abilities to form covalent and ionic bonds. The value of each property for each metal ion is provided in Supplemental Material, Table S1 (http://dx.doi.org/10.1289/ehp.1205793). In addition to evaluating associations with the properties of the individual metal ions, we evaluated associations according to two groups of metal ions. The *s*-block group comprised Li, Na, K, Cs, Mg, Ca, and Ba, which are from the *s*-block of the periodic table. The transition group included the transition metal ions Mn, Fe, Co, Ni, Ag, Cu, Zn, and Hg from the *d*-block of the periodic table, which can have multiple oxidation states. Although Pb is from the *p*-block of the periodic table, it was included with the transition metal ions for this analysis because it also has multiple oxidation states.

**Table 1:** The ROS generation–related properties of metal ions used in the analysis.

Abbreviation	Description	Property affecting ROS generationa
AN	Atomic number	Ion mass
AR	Atomic radius	Ion length scale
r	Pauling ionic radius	Ion length scale
ρ	Density	Ion mass and length scale
ΔE0	Change in electrochemical potential from ion to its first reduced state	Tendency of an ion to be oxidized
ΔIP	Change in ionization potential from ion to its first reduced state	Energy required for oxidation
Z	Ion charge	Oxidation state
AN/ΔIP	Atomic number:ionization potential ratio	Oxidation energy
log(KOH)	Logarithm of the first hydrolysis constant	Ability to produce hydroxyl ions
MP	Melting point	Solubility
pKsp(CO3)	Solubility product of MCO3, where M = metal	Solubility
σp	Pearson softness coefficient	Softness
Xm	Electronegativity	Ability to form covalent bonds
Xm2r	Covalent index	Ability to form covalent bonds
Z/AR	Ionic charge:atomic radius ratio	Ability to form ionic bonds
Z2/r	Cation polarizing power	Ability to form ionic bonds
CO3, carbonate. aData from McCloskey et al. (1996), Mendes et al. (2010), Walker et al. (2003), and Wolterbeek and Verberg (2001).		

Associations of CVDs with metal ion exposures examined in this analysis came from the publicly available Comparative Toxicogenomics Database (CTD; Davis et al. 2009, 2011). The CTD is a curated database of known interactions between chemicals and genes, genes and diseases, and infrequently, chemicals and diseases. Chemical–disease relationships are inferred in the CTD using established evidence of either chemical × gene and gene × disease interactions or chemical × disease interactions observed in curated laboratory studies within the CTD database. Chemical × gene or chemical × disease interactions curated in the CTD can include any study that demonstrates a chemical exposure can lead to a change in gene or disease status through any exposure pathway (Davis et al. 2009, 2011). The CTD database focuses on environmental chemicals and outcomes relevant to human health, but inferences may be based in part on information from animal studies if the animal contains a gene that is also present in humans and the study elucidates the effect of a chemical on the gene or a gene on a disease outcome.

Within the CTD, inference score is a measure of the degree of support for a given association between a disease and a chemical. An inference score is computed using principles of scale-free networks, where the probability (*P*) that a vertex in a large network interacts with another vertex decays according to a power law (Barabasi and Albert 1999). A vertex in the CTD can be a gene (*G*), chemical (*C)*, or disease (*D*) with some number of connections (*k*) between them. For a set of *n_G_* genes, the inference score (*Y*) is computed as shown below (Li and Liang 2009):

*Y* = –ln[*P*(*G* associated with both *C* and *D*|*k*, *n_G_*)*P*(no other *G* connects *C* and *D*|*k*, *n_G_*)]. [1]

Each inference score used in this analysis is the log transform of the product of two probability functions: *a*) the probability that a gene is associated with both a chemical and a disease, and *b*) the probability that the chemical–gene–disease connection is unique. The CTD curates data for chemical × disease and gene × disease interactions for 27 disease categories, including CVDs. Within the CVD category, five end points were selected for this study based on the potential relevance of oxidative stress mechanisms to their development: cardiac arrhythmia, myocardial ischemia, myocardial infarction, stroke, and thrombosis (Godleski et al. 2000; Longhurst et al. 2001; U.S. EPA 2009). Inference score data for each CVD end point–metal ion pair is provided in Supplemental Material, Table S2 (http://dx.doi.org/10.1289/ehp.1205793). Arrhythmia inference scores were available for 6 of 7 *s*-block metal ions and 7 of 10 transition metal ions, myocardial infarction inference scores were available for 5 *s*-block metal ions and 8 transition metal ions, myocardial ischemia inference scores were available for 5 *s*-block metal ions and 9 transition metal ions, and thrombosis and stroke inference scores were available for 5 *s*-block metal ions and 10 transition metal ions.

*Statistical analysis*. All statistical analyses were conducted with SAS version 9.1 (SAS Institute Inc., Cary, NC). For each CVD end point, least-trimmed squares (LTS) regression was used to estimate associations of log-transformed inference scores for a set of metal ions (*s*-block or transition) with their ion properties. LTS regression minimizes the influence of outliers on the model fit (Rousseeuw and Hubert 2011; Rousseeuw and Leroy 2003). Rather than minimize the sum of squared residuals for all data points included in ordinary least squares regression, LTS minimizes the sum of squared residuals for a subset of data points that minimizes the sum of squared residuals to remove the influence of outliers from the regression. Trimmed data points are those data points excluded from the minimized sum of squared residuals function, and the number of trimmed data points (*n*_trim_) is determined separately for each outcome and metal ion group model. The default approach provided by SAS (PROC ROBUSTREG) was employed to calculate LTS breakdown values to determine *n*_trim_. Breakdown values estimate the smallest proportion of data that, if erroneous, could bias the estimator. Breakdown values for the simulations ranged from 8 tto 20%.

The overall significance level was 0.05. Given that 16 properties were tested, the multiple comparisons design led to a Bonferroni-corrected significance level of 0.003 (or 0.05/16). Log transformation of the inference scores was applied to reduce heteroscedascity and more closely meet the statistical modeling assumptions.

**Figure 2:**
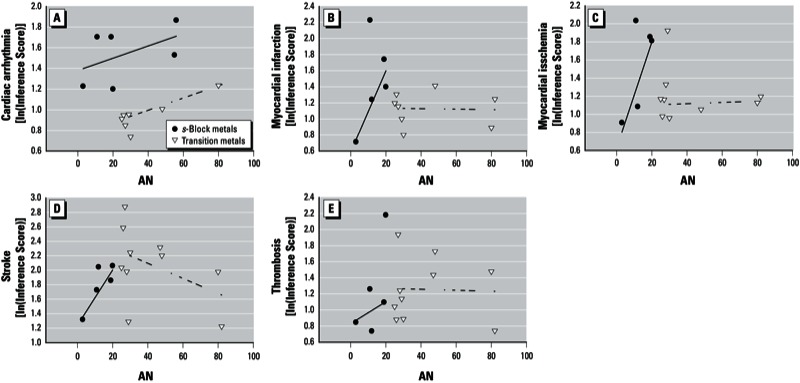
Scatter plots of ln(Inference Score) vs. AN for each CV end point. Arrhythmia (*A*), myocardial infarction (*B*), myocardial ischemia (*C*), stroke (*D*), and thrombosis (*E*). The solid lines represent LTS robust regressions for the s-block metal ions, and the dashed lines represent LTS robust regressions for the transition metal ions. All data points (trimmed and nontrimmed) are shown in the figure with the robust regression lines.

Given collinearity between several ion properties, only one ion property was used in a univariate regression each time as a predictor (Table 2). Scatter plots were developed during exploratory analysis to visualize the data [Figure 2 shows an example for atomic number (AN)]. Examination of these scatter plots revealed that the properties tended to cluster around the type of metal ion (i.e., *s*-block or transition), with potentially different slopes for each group. Therefore, LTS regressions for each disease–property combination were performed separately for *s*-block and transition metal ions. Slopes obtained from the LTS regressions are presented both as 1-unit changes in properties and as standardized by the interquartile range (IQR) of the property distribution.

**Table 2:** Spearman correlation coefficients among the physical and chemical properties across the metal ions included in the QICAR models.

	Z	AN	r	AR	ΔIP	ΔE0	Xm	log(KOH)	σp	Xm2r	Z2/r	AN/ΔIP	Z/AR	MP	ρ	pKsp(CO3)
Z	1.0	0.24	–0.47	–0.49	0.50	–0.50	0.48	–0.57	–0.54	0.26	0.88	–0.04	0.86	0.49	0.46	–0.45
AN		1.0	0.48	0.13	–0.03	–0.32	0.43	–0.36	–0.50	0.71	–0.07	0.85	0.15	–0.25	0.62	–0.58
r			1.0	0.89	–0.71	0.50	–0.47	0.52	0.35	–0.04	–0.75	0.82	–0.69	–0.55	–0.27	–0.09
AR				1.0	–0.75	0.80	–0.78	0.67	0.65	–0.44	–0.71	0.58	–0.81	–0.55	–0.65	0.23
ΔIP					1.0	–0.77	0.71	–0.83	–0.74	0.34	0.77	–0.42	0.73	0.67	0.60	–0.33
ΔE0						1.0	–0.95	0.74	0.85	–0.74	–0.61	0.14	–0.80	–0.48	–0.90	0.66
Xm							1.0	–0.78	–0.85	0.87	0.55	–0.07	0.76	0.39	0.94	–0.63
log(KOH)								1.0	0.80	–0.56	–0.67	0.10	–0.74	–0.32	–0.76	0.46
σp									1.0	–0.71	–0.54	–0.06	–0.66	–0.35	–0.91	0.73
Xm2r										1.0	0.16	0.30	0.47	0.06	0.86	–0.65
Z2/r											1.0	–0.39	0.92	0.66	0.44	–0.31
AN/ΔIP												1.0	–0.26	–0.42	0.16	–0.26
Z/AR													1.0	0.59	0.67	–0.53
MP														1.0	0.30	–0.34
ρ															1.0	–0.74
pKsp(CO3)																1.0

To evaluate whether the results were sensitive to specific regression methods, additional regression approaches were also used to examine the robustness of the LTS results. Other regression approaches included in the analysis were maximum likelihood type robust (M) estimation (Huber 1973), minimization of robust scale (S) estimation (Rousseeuw and Yohai 1984), and high breakdown/high efficiency robust (MM) estimation (Yohai 1987). For M estimation, a bi-square weight function was applied. For MM and S estimations, the SAS default parameters in PROC ROBUSTREG for the MM and S options, respectively, were employed.

Because the data set had small sample sizes (i.e., it was limited to the number of metal ions included in each group evaluated), robustness of the LTS results was further evaluated via Monte Carlo simulation. The log-transformed inference scores, *Y_ij_*, were sampled once from the corresponding normal distribution with a mean of β_0_*_,ikl_* + β_1_*_,ikl_x_jk_* and a standard deviation of σ*_ikl_* for the *i*th disease, *j*th metal ion, *k*th metal ion property, and *l*th group (*s*-block vs. transition metal ions). β_0_ and β_1_ are the regression coefficients (treated as fixed values in the simulation) between log-transformed inference scores and metal ion properties, *x* is the metal ion property (treated as a fixed value in the simulation), and σ is the standard deviation of the regression residuals. The sampled *Y_ij_* were regressed on *x_jk_* with the LTS approach for each combination of health end points, metal ion properties, and metal ion groups. The number of outliers [defined as *Y_ij_* = (β_0_*_,ikl_ +* β_1_*_,ikl_x_jk_*) ± 3σ*_ikl_* ] included in each simulated data set was greater than the number of outliers identified by LTS in the original data set. The procedure was repeated 1,000 times. Centrality and variability of the regression coefficients based on the original data sets were compared with the simulation results.

*Model validation*. Validation of selected models was performed where data were available in the CTD and properties databases. Because there are more transition metal ions than *s*-block metal ions, four cardiac arrhythmia models were chosen for validation: AN, σ_p_, melting point (MP), and density (ρ) (Dean 1999; Hammond 2003; James and Lord 1992; Kaye and Laby 1993). Relative *L*_2_ error norms were computed to compare ln(Inference Score) predictions from the regression models input with property data for aluminum [Al(III)], chromium [Cr(VI)], and vanadium [V(V)]. In other words, the relative *L*_2_ error norm approaches zero as the predicted ln(Inference Score) approaches that from the CTD. Relative *L*_2_ error norm is computed as


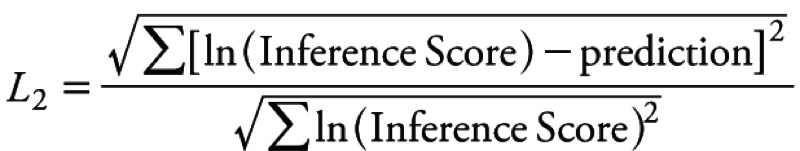
[2]

## Results and Discussion

All associations between inference scores and ion properties (robust univariate regressions) are summarized [see Supplemental Material, Tables S3–S7 (http://dx.doi.org/10.1289/ehp.1205793)]. A set of example scatter plots with LTS regression lines is provided for the AN property in Figure 2. Table 3 presents significant LTS regression results, with *p*-values of the slopes < 0.003. Table 3 also provides data for the number of metal ions included and trimmed from the robust model, SE, significance level, and coefficient of determination (*R*^2^). Table 4 displays a matrix illustrating statistically significant associations between inference scores and properties for each metal ion grouping (*s*-block or transition metal ions).

**Table 3:** The associations between the inference score relating CVD to metal exposures with the physical and chemical properties of metal ions using robust univariate regressions.

Disease	Group	Property	na	ntrimb	Slope	Standardized slopec	SE	p-Value	R2
Cardiac arrhythmia	Transition	Xm2r	6	1	0.177	0.112	0.0506	< 0.001	0.48
		AN	6	1	5.86 × 10–3	2.79 × 10–4	8.95 × 10–4	< 0.001	0.84
		σp	6	1	–5.54	11.1	1.76	0.002	0.7
		ρ	6	1	6.27 × 10–5	3.09 × 10–8	1.53 × 10–5	< 0.001	0.59
		MP	6	1	–1.81 × 10–4	–1.72 × 10–7	4.84 × 10–5	< 0.001	0.57
Myocardial infarction	s-Block	AN/ΔIP	4	1	0.235	0.0375	0.054	< 0.001	0.9
		AN	4	1	0.0504	1.62 × 10–3	0.0147	0.001	0.85
Myocardial ischemia	s-Block	Xm2r	4	1	–2.6	–17.3	0.262	< 0.001	0.98
		AN/ΔIP	4	1	0.276	0.0441	0.0416	< 0.001	0.94
		r	4	1	3.3	8.24	0.514	< 0.001	0.92
		AR	4	1	2.59	6.46	0.519	< 0.001	0.87
		AN	4	1	0.0584	1.89 × 10–3	0.0151	< 0.001	0.84
		Xm	4	1	–1.82	–18.2	0.567	0.001	0.84
Stroke	s-Block	AN	4	1	0.0381	1.23 × 10–3	7.35 × 10–3	< 0.001	0.73
		MP	4	1	3.73 × 10–4	5.74 × 10–7	1.14 × 10–4	1.09 × 10–3	0.84
		σp	4	1	–9.63	–241	1.33	< 0.001	0.83
		Xm2r	4	1	2.53	16.9	0.0828	< 0.001	0.99
		Z	4	1	0.260	0.260	0.0663	< 0.001	0.88
Thrombosis	s-Block	ρ	4	1	0.36 × 10–3	1.20	1.56 × 10–4	< 0.001	0.84
		σp	4	1	–19.7	–492	3.71	< 0.001	0.60
	Transition	AR	8	2	2.21	11.0	0.544	< 0.001	0.66
		σp	7	2	–10.2	–204	3.01	< 0.001	0.68
		Z/AR	8	2	–2.60	–11.3	0.572	< 0.001	0.65
Results reported are for the models with significant slopes (p-values < 0.003). Properties are listed in descending order of R2 within each disease and metal ion group. aNumber of metals included in model. bNumber of metals trimmed from the robust model based on total number for which data were available. cStandardized per IQR increase in property. For complete results, see Supplemental Material, Tables S3–S7 (http://dx.doi.org/10.1289/ehp.1205793).									

**Table 4:** The statistically significant associations (*p* < 0.003) between the CTD inference score relating CVD to metal exposures, and the physical and chemical properties of metal ions, by the groups of metal ions (s-block and transition metal ions).

Property	Cardiac arrhythmia	Myocardial infarction	Myocardial ischemia	Stroke	Thrombosis
AN	Transitiona	s-Block	s-Block	s-Block	—
AR	—	—	s-Block	—	Transition
ρ	Transition	—	—	—	s-Block
MP	Transition	—	—	s-Block	—
r	—	—	s-Block	—	—
σp	Transition	—	—	s-Block	s-Block, transition
Xm	—	—	s-Block	—	—
AN/ΔIP	—	s-Block	s-Block	—	—
Xm2r	Transition	—	s-Block	s-Block	—
Z	—	—	—	s-Block	—
Z/AR	—	—	—	—	Transition
—, No statistically significant relationship between the health outcome and property. aSignificant associations (p-values < 0.003 for slopes and R2 > 0.5 in the robust univariate regression) between cardiac arrhythmia and AN for transition metal ions.					

Evaluation of the LTS regression results through comparison with other robust estimation methods and Monte Carlo simulations to address small sample size lend confidence to the results. LTS regression produced slopes that were often comparable to those associated with the other robust estimation approaches [see Supplemental Material, Tables S8–S12 (http://dx.doi.org/10.1289/ehp.1205793)], with equivalent slopes produced by the four methods for 50% of the simulations and slopes within 20% of each other for 63% of the simulations. The Monte Carlo simulation results also confirmed the robustness of slopes (see Supplemental Material, Tables S13–S17) by predicting the model slopes within ± 3%, although the SE of the LTS results tended to underestimate the SE computed during the Monte Carlo simulations. The LTS approach is very robust to outliers (Nevitt and Tam 1998; You 1999).

The validation exercise produced *L*_2_ error norms (i.e., the relative difference between the predicted inference score and the CTD inference score) for AN, σ_p_, MP, and ρ of 29%, 14%, 25%, and 41%, respectively. These *L*_2_ error norms are high, but given that data from only three metals were used to validate the models, they suggest a reasonable fit.

Associations between health outcomes and properties varied among the diseases and metal ion groupings. Cardiac arrhythmia was not associated with any properties for the *s*-block elements (Tables 3 and 4). For the transition elements, cardiac arrhythmia was associated with ion mass and length scale (AN and ρ), solubility (MP), softness (σ_p_), and the ability to form covalent bonds (X_m_^2^r). Myocardial infarction was associated with two properties for the *s*-block element models and none for the transition metal ion models: ion mass (AN) and oxidation energy (AN/ΔIP). Myocardial ischemia was associated with six properties for the *s*-block metal ion models and none for the transition metal ion models. For the *s*-block metal ions, associations were observed for ion size [AN, AR, r], oxidation energy (AN/ΔIP), and ability to form covalent bonds (X_m_, X_m_^2^r). Stroke was associated with five properties for the *s*-block metal ion models and none for the transition metal ion models. For the *s*-block metal ions, associations were observed for ion mass (AN), solubility (MP), softness (σ_p_), ability to form covalent bonds (X_m_^2^r), and ability to form ionic bonds (Z). Thrombosis was associated with two properties for the *s*-block metal ions, ion mass and length scale (ρ) and softness (σ_p_), and three properties for the transition metal ions, ion length scale (AR), softness (σ_p_), and the ability to form ionic bonds (Z/AR). Thrombosis was the only end point for which both the *s*-block and transition metal ions had significant models.

Similarities and differences among the regression models are apparent for the health end points. The slopes, based on unit and IQR changes, were similar in magnitude and sign for myocardial ischemia and myocardial infarction for AN, AN/ΔIP, log(*K*_OH_), and ρ [see Supplemental Material, Tables S4 and S5, respectively (http://dx.doi.org/10.1289/ehp.1205793)]. For example, the *s*-block model unit slopes for AN were 0.0504 and 0.0584 for myocardial infarction and myocardial ischemia, respectively. These observations, along with the finding that associations of myocardial ischemia and myocardial infarction were observed primarily for *s*-block elements (Table 3), suggest a common mechanistic pathway for both outcomes among the metal ions examined. Myocardial infarction is a severe potential consequence of myocardial ischemia, to which altered vasoreactivity, atherosclerotic plaque formation, and thrombosis may contribute (Figure 1). Estimated slopes for associations between cardiac arrhythmia and metal ion properties tended to differ from estimated slopes for other outcomes. For example, slopes for AN, AN/ΔIP, and ρ varied in magnitude between cardiac arrhythmia and myocardial ischemia [see Supplemental Material, Tables S3 and S5, respectively (http://dx.doi.org/10.1289/ehp.1205793)]. The *s*-block model unit slope for AN was an order of magnitude lower for cardiac arrhythmia (0.00583) compared with myocardial infarction (0.0504) and myocardial ischemia (0.0584). It is possible that these observed differences reflect the role of autonomic nervous system imbalance in the development of cardiac arrhythmia (see also Campese et al. 2004; Creason et al. 2001; Gold et al. 2000; He et al. 2010, 2011; Liao et al. 2009, 2010; Pope et al. 1999). Differences were observed between the statistically significant property-based models for thrombosis and stroke. It is possible that differences among the stroke and thrombosis models may relate to etiologic differences between strokes of hemorrhagic, rather than thromboembolic, origin. Previous epidemiologic studies have reported associations between PM, nitrogen dioxide, carbon monoxide, and ozone with ischemic (i.e., thromboembolic) but not hemorrhagic stroke (Hong et al. 2002; Wellenius et al. 2005). However, the models for softness (σ_p_) were statistically significant for stroke with *s*-block metal ions and for thrombosis with both *s*-block and transition metal ions.

Associations between CVD and transition metal ion exposure have been found in toxicological and epidemiologic studies. Farraj et al. (2011) exposed rats to a PM designed to mimic metal ion–containing residual oil fly ash by composing the PM of a mix of NiSO_4_ (nickle sulfate), Fe_2_SO_4_ [iron(II) sulfate], and NaVO_3_ [sodium vanadate(V)] and observed cardiac arrhythmias with concurrent autonomic changes, with the magnitude of autonomic change corresponding to low, medium, or high exposure group and no effect for the no exposure group. Given that Farraj et al. (2011) studied only transition metal ions, this is somewhat consistent with the findings that the transition metal ion properties were significantly associated with cardiac arrhythmia. However, Farraj et al. (2011) did not look at associations between cardiac arrhythmia and individual metal ion compounds, so it is not possible to discern whether relative differences among the properties had differential effects on arrhythmogenesis. An epidemiologic study of hospital admissions for CVD among older adults (> 64 years of age) in Atlanta, Georgia, by Suh et al. (2011) reported significant associations between myocardial ischemia and transition metals in PM exposure, but did not study cardiac arrhythmias. However, our results indicate that for the transition metal ions, myocardial ischemia is not statistically significantly associated with any other properties examined. Hence, the results do not provide strong support for or negation of Suh et al.’s (2011) observations. Based on the results of the QICAR models, none of the CV health outcomes were associated with ionic exchange of electrons for transition metal ions, in contrast with with *s*-block metal ions, which were associated with myocardial ischemia and myocardial infarction. However, all five adverse CV health outcomes were associated with ion size for both transition and *s*-block metal ions. Ion size or mass may reflect greater reactivity, possibly resulting from stronger interatomic forces associated with complexes involving large ions (Walker et al. 2003). Softness (σ_p_) was found to be a statistically significant predictor of cardiac arrhythmia for transition metal ions (Table 3). Mendes et al. (2010) concluded that σ_p_ caused covalent bonding of the metal ions to biological ligands in a study of fungi toxicity. σ_p_ also provided the most statistically significant association with adverse species outcomes in several other ecotoxicology studies (McCloskey et al. 1996; Ownby and Newman 2003; Zhou et al. 2011).

There are several limitations of this work. First, the sample sizes were small because the number of *s*-block and transition metal ions is small in general, and only a subset is available in the CTD. Small sample size also limits the model validation, given that data from only three metal ions were available for validation. However, our analysis was intended to explore development of models potentially associating adverse CV end points and chemical and physical properties, rather than to establish definitive conclusions about these relationships. Furthermore, Monte Carlo simulations yielded slope estimates within ± 3%, which added confidence to the results. Second, the properties evaluated were moderately to highly correlated (Table 2), thus limiting the linear regression analyses to univariate regressions. More sophisticated multivariate approaches not constrained by collinearity (e.g., partial least square regressions) need to be tested to examine further the associations between disease outcomes and ion properties. Third, the CTD is likely subject to selection bias because the inference scores reflect both data availability and inference of association between each chemical–disease pair in question. Moreover, given that the CTD inferred associations between disease outcomes and chemical exposures, the CTD may not have been limited to inhalation exposures, which is the primary exposure pathway for air pollutants. However, the inference score calculated within the CTD was attractive to use as a health outcome metric because it incorporates probabilities of association in a consistent manner across health outcomes and outcome–property pairs. These limitations will be addressed as the methodology of applying QICAR to human health outcomes is refined.

## Conclusions

In this exploratory, hypothesis-generating work, we used QICAR to link human CVD and the properties of metal ions commonly observed in ambient PM. Cardiac arrhythmia, myocardial infarction, myocardial ischemia, stroke, and thrombosis were associated with some ion properties related to ROS generation. This work supports the feasibility of using ion properties to predict CVD. QICAR has the potential to complement existing epidemiologic methods for estimating associations between CVDs and air pollutant exposures by providing clues about the underlying mechanisms that may explain these associations. More sophisticated approaches will be applied to extend work to study the associations between diseases and properties of organic and inorganic chemicals.

## Supplemental Material

(840 KB) PDF
