# Supplementary material for: Cardiovascular Outcomes and the Physical and Chemical Properties of Metal Ions Found in Particulate Matter Air Pollution: A QICAR Study
Source: Environ Health Perspect. 2013 Mar 5;121(5):558–64. doi: 10.1289/ehp.1205793 (PMC3673192; doi:10.1289/ehp.1205793)
Supplement: (840 KB) PDF [file ehp.1205793.s001.pdf]

Supplemental Material

Cardiovascular Outcomes and the Physical and Chemical Properties of Metal Ions Found in Particulate Matter Air Pollution: a QICAR Study

Qingyu Meng, Jennifer Richmond-Bryant, Shou-En Lu, Barbara Buckley, William J. Welsh, Eric A. Whitsel, Adel Hanna, Karin B. Yeatts, Joshua Warren, Amy H. Herring, Aijun Xiu

Table of Contents

Table S1. Properties of metal ions included in the QICAR analysis.	S3
Table S2. CTD inference scores, a measure of the degree of support for a given association between a disease and a chemical based on known chemical-gene and gene-disease interactions, for metal ions included in the QICAR analysis, by CVD endpoint.	S4
Table S3. The associations between inference score relating CVD to metal exposures with the physical and chemical properties of metal ions using robust univariate regressions for cardiac arrhythmia.	S5
Table S4. The associations between inference score relating CVD to metal exposures with the physical and chemical properties of metal ions using robust univariate regressions for myocardial infarction.	S6
Table S5. The associations between inference score relating CVD to metal exposures with the physical and chemical properties of metal ions using robust univariate regressions for myocardial ischemia.	S7
Table S6. The associations between inference score relating CVD to metal exposures with the physical and chemical properties of metal ions using robust univariate regressions for thrombosis.	S8
Table S7. The associations between inference score relating CVD to metal exposures with the physical and chemical properties of metal ions using robust univariate regressions for stroke.	S9
Table S8. Comparisons of regression results from the various robust regression methods for cardiac arrhythmia.	S10
Table S9. Comparisons of regression results from the various robust regression methods for myocardial infarction.	S11
Table S10. Comparisons of regression results from the various robust regression methods for myocardial ischemia.	S12
Table S11. Comparisons of regression results from the various robust regression methods for stroke.	S13
Table S12. Comparisons of regression results from the various robust regression methods for thrombosis.	S14
Table S13. Average results from 1000 Monte Carlo simulations of the LTS regression. Percent error denotes the relative error between the LTS slope from the data model fit compared with the LTS slope from the Monte Carlo simulation for cardiac arrhythmia.	S15
Table S14. Average results from 1000 Monte Carlo simulations of the LTS regression. Percent error denotes the relative error between the LTS slope from the data model fit compared with the LTS slope from the Monte Carlo simulation for myocardial infarction.	S16
Table S15. Average results from 1000 Monte Carlo simulations of the LTS regression. Percent error denotes the relative error between the LTS slope from the data model fit compared with the LTS slope from the Monte Carlo simulation for myocardial ischemia.	S17
Table S16. Average results from 1000 Monte Carlo simulations of the LTS regression. Percent error denotes the relative error between the LTS slope from the data model fit compared with the LTS slope from the Monte Carlo simulation for stroke.	S18
Table S17. Average results from 1000 Monte Carlo simulations of the LTS regression. Percent error denotes the relative error between the LTS slope from the data model fit compared with the LTS slope from the Monte Carlo simulation for thrombosis.	S19
References	S20

Table S1. Properties of metal ions included in the QICAR analysis.

Metal	Z	AN	r	AR	ΔIP	ΔE^0	X_m	$\log(K_{OH})$	σ_p	X_m^2r	Z^2/r	AN/ΔIP	Z/AR	MP	P	$pKsp(CO_3)$
s-block Metals																
Li (I)	1	3	0.7	1.5	5.39	3.05	1	13.6	0.25	0.71	1.35	0.56	0.66	453.69	535	-1.60
Na (I)	1	11	1	1.9	5.14	2.71	0.9	14.2	0.21	0.88	0.98	2.14	0.54	370.87	968	NA
K (I)	1	19	1.4	2.3	4.34	2.92	0.8	14.5	0.23	0.93	0.72	4.38	0.43	336.53	856	NA
Cs (I)	1	55	1.7	2.6	3.89	2.92	0.8	14.9	0.22	1.06	0.59	14.14	0.38	301.59	1879	-8.47
Mg (II)	2	12	0.7	1.6	7.39	2.38	1.3	11.6	0.17	1.24	5.56	1.62	1.29	923	1738	-7.46
Ca (II)	2	20	1	1.9	5.76	2.76	1	12.7	0.18	1	4	3.47	1.05	1115	1550	-8.42
Ba (II)	2	56	1.4	2.2	4.79	2.9	0.9	13.4	0.18	1.08	2.92	11.69	0.92	1000	3510	-8.29
Transition Metals																
Mn (II)	2	25	0.8	1.3	8.21	1.03	1.6	10.6	0.13	1.99	4.82	3.04	1.53	1519	7470	-10.74
Fe (II)	2	26	0.6	1.2	16.2	0.45	1.8	2.04	NA	2.04	6.55	1.61	1.61	1811	7874	-10.49
Co (II)	2	27	0.8	1.1	9.19	0.28	1.9	9.7	0.13	2.65	5.33	2.94	1.79	1768	8900	-12.85
Ni (II)	2	28	0.7	1.3	10.5	0.23	1.9	9.9	0.13	2.52	5.8	2.66	1.6	1728	8908	-8.18
Ag (I)	1	47	1.2	1.5	7.58	0.8	1.9	12	0.07	4.28	0.87	6.21	0.66	1234.9	10490	-11.09
Cu (II)	2	29	0.7	1.4	12.6	0.16	1.9	8	0.1	2.64	5.48	2.31	1.48	1357.8	8920	-9.85
Zn (II)	2	30	0.8	1.3	8.57	0.76	1.7	9	0.12	2.04	5.33	3.5	1.6	692.68	7140	-9.84
Cd (II)	2	48	1	1.5	7.91	0.4	1.7	10.1	0.08	2.71	4.21	6.07	1.35	594.22	8650	-11.28
Hg (II)	2	80	1	1.5	8.32	0.91	2	3.4	0.07	4.08	3.92	9.62	1.35	234.32	13534	NA
Pb (II)	2	82	1.2	1.5	7.61	0.13	2.3	7.7	0.13	6.41	3.39	10.78	1.61	600.61	11340	-13.13

NA: not available

Sources: Dean (1999); James and Lord (1992); Kaye and Laby (1993); Lide (2003); Mendes et al. (2010); Wolterbeek and Verberg (2001)

Table S2. CTD inference scores, a measure of the degree of support for a given association between a disease and a chemical based on known chemical-gene and gene-disease interactions, for metal ions included in the QICAR analysis, by CVD endpoint.

Metal	Cardiac Arrhythmia	Myocardial Infarction	Myocardial Ischemia	Thrombosis	Stroke
s-block Metals					
Li (I)	3.41	2.04	2.48	2.33	3.74
Na (I)	5.49	9.27	7.64	3.52	5.62
K (I)	5.49	5.7	6.39	2.99	6.41
Cs (I)	4.61	NA	NA	NA	NA
Mg (II)	NA	3.46	2.96	2.09	7.72
Ca (II)	3.32	4.05	6.12	8.85	7.85
Ba (II)	6.45	NA	NA	NA	NA
Transition Metals					
Mn (II)	2.49	3.31	3.22	2.83	7.64
Fe (II)	2.58	3.69	2.66	2.41	13.29
Co (II)	2.34	3.18	3.2	6.95	17.76
Ni (II)	NA	NA	3.78	3.46	7.24
Ag (I)	NA	NA	NA	4.2	10.15
Cu (II)	2.6	2.72	6.85	3.12	3.63
Zn (II)	2.09	2.23	2.61	2.43	9.41
Cd (II)	2.74	4.11	2.87	5.63	9.03
Hg (II)	3.44	2.44	3.09	4.38	7.22
Pb (II)	NA	3.48	3.31	2.1	3.4

NA: inference scores were not available in the CTD for this disease-metal combination.
Source: The Comparative Toxicogenomics Database. http://ctdbase.org. Accessed 21 February, 2013.

Table S3. The associations between inference score relating CVD to metal exposures with the physical and chemical properties of metal ions using robust univariate regressions for cardiac arrhythmia.

Property	Group	N[a]	N_{trim}[b]	Intercept	Slope[c]	Standardized Slope[d]	SE	Lower CL	Upper CL	P-Value	R^2
AN	s-block	5	1	1.38E+00	5.86E-03	1.89E-04	5.25E-03	-4.43E-03	1.62E-02	2.64E-01	2.48E-01
AN	transition	6	1	7.55E-01	5.86E-03	2.79E-04	8.95E-04	4.10E-03	7.61E-03	5.97E-11	8.35E-01
AN/ΔIP	s-block	5	1	1.40E+00	2.25E-02	3.60E-03	2.20E-02	-2.07E-02	6.56E-02	3.07E-01	1.66E-01
AN/ΔIP	transition	6	1	7.74E-01	4.23E-02	1.23E-02	1.58E-02	1.13E-02	7.33E-02	7.52E-03	6.08E-01
AR	s-block	5	1	8.21E-01	3.47E-01	8.67E-01	3.09E-01	-2.58E-01	9.52E-01	2.61E-01	2.36E-01
AR	transition	6	1	-2.04E-01	8.75E-01	4.37E+00	3.47E-01	1.94E-01	1.56E+00	1.18E-02	3.99E-01
ΔE^0	s-block	5	1	2.59E+00	-3.65E-01	-2.15E+00	1.09E+00	-2.51E+00	1.78E+00	7.38E-01	4.02E-01
ΔE^0	transition	6	1	9.58E-01	-1.09E-01	-1.98E-01	1.38E-01	-3.79E-01	1.61E-01	4.29E-01	1.35E-01
ρ	s-block	5	1	1.34E+00	1.28E-04	1.13E-07	1.09E-04	-8.60E-05	3.42E-04	2.42E-01	4.03E-01
ρ	transition	6	1	3.89E-01	6.27E-05	3.09E-08	1.53E-05	3.27E-05	9.27E-05	4.14E-05	5.90E-01
ΔIP	s-block	5	1	2.61E+00	-2.19E-01	-2.71E-01	1.64E-01	-5.41E-01	1.03E-01	1.82E-01	4.31E-01
ΔIP	transition	6	1	8.13E-01	8.50E-03	3.86E-03	1.40E-02	-1.89E-02	3.59E-02	5.43E-01	8.46E-02
$\log(K_{OH})$	s-block	5	1	-5.04E-01	1.47E-01	1.23E-01	1.53E-01	-1.53E-01	4.47E-01	3.37E-01	5.99E-01
$\log(K_{OH})$	transition	6	1	1.13E+00	-2.36E-02	-1.04E-02	1.71E-02	-5.71E-02	9.83E-03	1.66E-01	3.29E-02
MP	s-block	5	1	1.61E+00	-1.20E-04	-1.80E-07	3.72E-04	-8.40E-04	6.12E-04	7.54E-01	4.70E-01
MP	transition	6	1	1.20E+00	-1.80E-04	-1.70E-07	4.84E-05	-2.80E-04	-8.60E-05	1.89E-04	5.70E-01
$pK_{sp}(CO_3)$	transition	5	1	1.28E+00	3.13E-02	2.19E-02	2.36E-02	-1.50E-02	7.75E-02	1.86E-01	3.69E-01
r	s-block	5	1	1.01E+00	4.36E-01	1.09E+00	3.31E-01	-2.13E-01	1.08E+00	1.88E-01	4.31E-01
r	transition	6	1	3.60E-01	7.32E-01	2.44E+00	3.66E-01	1.53E-02	1.45E+00	4.53E-02	1.14E-01
σ_p	s-block	5	1	3.43E+00	-8.37E+00	-2.09E+02	3.25E+00	-1.47E+01	-2.01E+00	9.97E-03	6.89E-01
σ_p	transition	5	1	1.52E+00	-5.54E+00	-1.11E+02	1.76E+00	-8.99E+00	-2.09E+00	1.67E-03	6.99E-01
X_m	s-block	5	1	3.27E+00	-1.93E+00	-1.93E+01	1.29E+00	-4.45E+00	6.01E-01	1.35E-01	4.59E-01
X_m	transition	6	1	-3.72E-02	5.53E-01	2.76E+00	3.52E-01	-1.38E-01	1.24E+00	1.17E-01	3.69E-02
X_m^2r	s-block	5	1	6.93E-01	8.95E-01	5.97E+00	8.87E-01	-8.44E-01	2.63E+00	3.13E-01	5.41E-01
X_m^2r	transition	6	1	4.90E-01	1.77E-01	1.12E-01	5.06E-02	7.78E-02	2.76E-01	4.73E-04	4.82E-01
Z	s-block	5	1	1.55E+00	-8.18E-03	-8.18E-03	2.64E-01	-5.26E-01	5.09E-01	9.75E-01	3.57E-01
Z/AR	s-block	5	1	1.91E+00	-7.08E-01	-1.47E+00	3.42E-01	-1.38E+00	-3.71E-02	3.86E-02	5.73E-01
Z/AR	transition	6	1	2.03E+00	-7.05E-01	-3.07E+00	3.07E-01	-1.31E+00	-1.04E-01	2.15E-02	3.71E-01
Z^2/r	s-block	5	1	1.64E+00	-5.63E-02	-2.06E-02	9.44E-02	-2.41E-01	1.29E-01	5.51E-01	4.90E-01
Z^2/r	transition	6	1	1.45E+00	-9.77E-02	-6.74E-02	6.50E-02	-2.25E-01	2.97E-02	1.33E-01	2.59E-02

[a]Number of metals included in model; [b]number of metals trimmed from the robust model based on total number for which data were available; [c]slope given per unit change in property; [d]standardized per interquartile range increase in property; SE: standard error; CL: 95% confidence limit.

Table S4. The associations between inference score relating CVD to metal exposures with the physical and chemical properties of metal ions using robust univariate regressions for myocardial infarction.

Property	Group	N[a]	N_{trim}[b]	Intercept	Slope[c]	Standardized Slope[d]	SE	Lower CL	Upper CL	P-Value	R^2
AN	s-block	4	1	5.94E-01	5.04E-02	1.62E-03	1.47E-02	2.16E-02	7.91E-02	6.07E-04	8.55E-01
AN	transition	6	2	1.14E+00	-3.30E-04	-1.60E-05	3.53E-03	-7.25E-03	6.60E-03	9.26E-01	1.03E-01
AN/ΔIP	s-block	4	1	6.85E-01	2.35E-01	3.75E-02	5.40E-02	1.29E-01	3.41E-01	1.40E-05	9.04E-01
AN/ΔIP	transition	6	2	1.13E+00	-7.30E-04	-2.10E-04	2.47E-02	-4.91E-02	4.77E-02	9.76E-01	1.20E-01
AR	s-block	4	1	-5.75E-01	1.01E+00	2.54E+00	3.92E-01	2.47E-01	1.78E+00	9.59E-03	7.70E-01
AR	transition	6	2	8.26E-01	2.23E-01	1.12E+00	5.88E-01	-9.29E-01	1.38E+00	7.04E-01	1.13E-01
ΔE^0	s-block	4	1	2.83E+00	-4.95E-01	-2.91E+00	1.26E+00	-2.96E+00	1.97E+00	6.93E-01	1.96E-01
ΔE^0	transition	6	2	1.24E+00	-2.29E-01	-4.17E-01	2.32E-01	-6.84E-01	2.25E-01	3.23E-01	3.37E-02
ρ	s-block	4	1	2.73E+00	-8.50E-04	-7.50E-07	3.91E-04	-1.61E-03	-8.00E-05	3.04E-02	6.89E-01
ρ	transition	6	2	1.29E+00	-1.80E-05	-8.70E-09	3.91E-05	-9.40E-05	5.89E-05	6.50E-01	2.27E-01
ΔIP	s-block	4	1	2.51E+00	-1.87E-01	-2.31E-01	2.69E-01	-7.14E-01	3.39E-01	4.85E-01	4.41E-01
ΔIP	transition	6	2	1.03E+00	9.54E-03	4.34E-03	2.83E-02	-4.60E-02	6.50E-02	7.36E-01	2.94E-02
$\log(K_{OH})$	s-block	4	1	-1.90E+00	2.68E-01	2.24E-01	1.26E-01	2.06E-02	5.16E-01	3.37E-02	6.80E-01
$\log(K_{OH})$	transition	6	2	1.07E+00	6.95E-03	3.06E-03	2.70E-02	-4.60E-02	5.99E-02	7.97E-01	2.59E-01
MP	s-block	4	1	2.27E+00	-9.10E-04	-1.40E-06	4.57E-04	-1.80E-03	-1.10E-05	4.73E-02	6.49E-01
MP	transition	6	2	1.01E+00	1.05E-04	1.00E-07	1.34E-04	-1.60E-04	3.68E-04	4.33E-01	5.38E-01
$pK_{sp}(CO_3)$	transition	6	1	3.69E-01	-7.08E-02	-4.95E-02	5.98E-02	-1.88E-01	4.63E-02	2.36E-01	5.10E-02
r	s-block	4	1	1.83E-01	1.32E+00	3.29E+00	9.51E-01	-5.46E-01	3.18E+00	1.66E-01	7.08E-01
r	transition	6	2	1.02E+00	1.20E-01	3.99E-01	4.62E-01	-7.86E-01	1.03E+00	7.96E-01	8.45E-02
σ_p	s-block	4	1	-4.62E-01	1.07E+01	2.67E+02	7.33E+00	-3.69E+00	2.51E+01	1.45E-01	4.96E-01
σ_p	transition	6	2	9.63E-01	1.29E+00	2.58E+01	3.69E+00	-5.94E+00	8.52E+00	7.27E-01	4.26E-01
X_m	s-block	4	1	2.65E+00	-1.18E+00	-1.18E+01	1.65E+00	-4.41E+00	2.06E+00	4.76E-01	4.23E-01
X_m	transition	6	2	1.07E+00	2.81E-02	1.40E-01	3.54E-01	-6.67E-01	7.23E-01	9.37E-01	1.92E-02
X_m^2r	s-block	4	1	3.96E+00	-2.28E+00	-1.52E+01	1.07E+00	-4.38E+00	-1.76E-01	3.36E-02	6.81E-01
X_m^2r	transition	6	2	1.09E+00	1.11E-02	7.04E-03	5.68E-02	-1.00E-01	1.22E-01	8.45E-01	4.49E-03
Z	s-block	4	1	2.65E+00	-6.64E-01	-6.64E-01	2.56E-01	-1.16E+00	-1.63E-01	9.42E-03	7.62E-01
Z/AR	s-block	4	1	2.39E+00	-8.86E-01	-1.85E+00	4.17E-01	-1.70E+00	-6.93E-02	3.35E-02	6.81E-01
Z/AR	transition	6	2	1.07E+00	3.95E-02	1.72E-01	5.84E-01	-1.11E+00	1.18E+00	9.46E-01	3.29E-01
Z^2/r	s-block	4	1	2.10E+00	-1.60E-01	-5.83E-02	6.59E-02	-2.89E-01	-3.07E-02	1.53E-02	7.36E-01
Z^2/r	transition	6	2	1.17E+00	-8.35E-03	-5.76E-03	8.51E-02	-1.75E-01	1.58E-01	9.22E-01	5.87E-02

[a]Number of metals included in model; [b]number of metals trimmed from the robust model based on total number for which data were available; [c]slope given per unit change in property; [d]standardized per interquartile range increase in property; SE: standard error; CL: 95% confidence limit.

Table S5. The associations between inference score relating CVD to metal exposures with the physical and chemical properties of metal ions using robust univariate regressions for myocardial ischemia.

Property	Group	N[a]	N_{trim}[b]	Intercept	Slope[c]	Standardized Slope[d]	SE	Lower CL	Upper CL	P-Value	R^2
AN	s-block	4	1	6.26E-01	5.84E-02	1.89E-03	1.51E-02	2.89E-02	8.80E-02	1.08E-04	8.40E-01
AN	transition	7	2	1.09E+00	6.56E-04	3.13E-05	2.02E-03	-3.31E-03	4.62E-03	7.46E-01	1.87E-01
AN/ΔIP	s-block	4	1	7.24E-01	2.76E-01	4.41E-02	4.16E-02	1.94E-01	3.57E-01	3.51E-11	9.41E-01
AN/ΔIP	transition	7	2	1.09E+00	5.49E-03	1.59E-03	1.43E-02	-2.25E-02	3.35E-02	7.01E-01	2.44E-01
AR	s-block	4	1	-2.96E+00	2.59E+00	6.46E+00	5.19E-01	1.57E+00	3.60E+00	6.42E-07	8.72E-01
AR	transition	7	2	1.07E+00	4.04E-02	2.02E-01	3.35E-01	-6.17E-01	6.98E-01	9.04E-01	8.40E-02
ΔE^0	s-block	4	1	-2.50E+00	1.56E+00	9.18E+00	6.97E-01	1.94E-01	2.93E+00	2.52E-02	7.15E-01
ΔE^0	transition	7	2	1.18E+00	-1.19E-01	-2.16E-01	1.41E-01	-3.95E-01	1.57E-01	3.98E-01	1.48E-02
ρ	s-block	4	1	2.66E+00	-7.50E-04	-6.70E-07	4.32E-04	-1.60E-03	9.39E-05	8.14E-02	6.03E-01
ρ	transition	7	2	9.55E-01	1.81E-05	8.93E-09	2.18E-05	-2.50E-05	6.09E-05	4.06E-01	2.48E-01
ΔIP	s-block	4	1	3.31E+00	-2.85E-01	-3.52E-01	1.08E-01	-4.97E-01	-7.28E-02	8.48E-03	7.76E-01
ΔIP	transition	7	2	1.24E+00	-1.28E-02	-5.80E-03	1.69E-02	-4.58E-02	2.03E-02	4.49E-01	2.91E-01
$\log(K_{OH})$	s-block	4	1	-1.84E+00	2.67E-01	2.22E-01	1.11E-01	4.88E-02	4.84E-01	1.64E-02	7.42E-01
$\log(K_{OH})$	transition	7	2	1.01E+00	1.45E-02	6.37E-03	1.40E-02	-1.30E-02	4.20E-02	3.01E-01	9.06E-02
MP	s-block	4	1	1.68E+00	-2.20E-04	-3.40E-07	8.12E-04	-1.81E-03	1.37E-03	7.88E-01	2.72E-01
MP	transition	7	2	1.07E+00	4.53E-05	4.31E-08	7.47E-05	-1.00E-04	1.92E-04	5.44E-01	3.56E-03
$pK_{sp}(CO_3)$	transition	6	2	3.78E-01	-6.22E-02	1.89E-03	2.36E-02	-1.09E-01	-1.59E-02	8.42E-03	6.34E-01
r	s-block	4	1	-1.41E+00	3.30E+00	3.13E-05	5.14E-01	2.29E+00	4.30E+00	1.46E-10	9.21E-01
r	transition	7	2	1.02E+00	1.25E-01	4.41E-02	2.58E-01	-3.81E-01	6.31E-01	6.28E-01	3.84E-01
σ_p	s-block	4	1	-3.59E-01	1.04E+01	1.59E-03	6.93E+00	-3.20E+00	2.40E+01	1.34E-01	5.29E-01
σ_p	transition	6	2	9.53E-01	1.72E+00	6.46E+00	1.80E+00	-1.81E+00	5.25E+00	3.40E-01	3.70E-01
X_m	s-block	4	1	3.55E+00	-1.82E+00	2.02E-01	5.67E-01	-2.93E+00	-7.12E-01	1.30E-03	8.38E-01
X_m	transition	7	2	7.52E-01	2.00E-01	9.18E+00	1.88E-01	-1.69E-01	5.69E-01	2.88E-01	2.14E-01
X_m^2r	s-block	4	1	4.33E+00	-2.60E+00	-2.16E-01	2.62E-01	-3.11E+00	-2.09E+00	2.88E-23	9.80E-01
X_m^2r	transition	7	2	1.04E+00	2.57E-02	-6.70E-07	3.12E-02	-3.54E-02	8.69E-02	4.09E-01	3.36E-01
Z	s-block	4	1	1.75E+00	-1.50E-01	8.93E-09	5.26E-01	-1.18E+00	8.80E-01	7.75E-01	4.68E-01
Z/AR	s-block	4	1	2.01E+00	-5.91E-01	-5.80E-03	7.28E-01	-2.02E+00	8.36E-01	4.17E-01	6.56E-01
Z/AR	transition	7	2	9.22E-01	1.29E-01	2.22E-01	3.37E-01	-5.31E-01	7.89E-01	7.02E-01	9.23E-03
Z^2/r	s-block	4	1	2.12E+00	-1.49E-01	6.37E-03	6.78E-02	-2.82E-01	-1.63E-02	2.78E-02	7.08E-01
Z^2/r	transition	7	2	1.24E+00	-2.35E-02	-3.40E-07	4.68E-02	-1.15E-01	6.83E-02	6.16E-01	4.03E-01

[a]Number of metals included in model; [b]number of metals trimmed from the robust model based on total number for which data were available; [c]slope given per unit change in property; [d]standardized per interquartile range increase in property; SE: standard error; CL: 95% confidence limit.

Table S6. The associations between inference score relating CVD to metal exposures with the physical and chemical properties of metal ions using robust univariate regressions for thrombosis.

Property	Group	N[a]	N_{trim}[b]	Intercept	Slope[c]	Standardized Slope[d]	SE	Lower CL	Upper CL	P-Value	R^2
AN	s-block	4	1	8.31E-01	1.36E-02	4.38E-04	2.36E-02	-3.28E-02	5.99E-02	5.66E-01	1.42E-01
AN	transition	8	2	1.28E+00	-6.42E-04	-3.10E-05	6.25E-03	-1.29E-02	1.16E-02	9.18E-01	3.30E-01
AN/ΔIP	s-block	4	1	8.18E-01	7.66E-02	1.22E-02	8.86E-02	-9.70E-02	2.50E-01	3.87E-01	2.72E-01
AN/ΔIP	transition	8	2	1.25E+00	-4.14E-04	-1.20E-04	4.32E-02	-8.51E-02	8.43E-02	9.92E-01	4.23E-01
AR	s-block	4	1	2.07E-01	4.29E-01	1.07E+00	3.39E-01	-2.36E-01	1.09E+00	2.06E-01	4.45E-01
AR	transition	8	2	-1.77E+00	2.21E+00	1.10E+01	5.44E-01	1.14E+00	3.27E+00	4.89E-05	6.62E-01
ΔE^0	s-block	4	1	3.61E-01	2.25E-01	1.33E+00	5.49E-01	-8.51E-01	1.30E+00	6.81E-01	7.77E-02
ΔE^0	transition	8	2	1.25E+00	-8.10E-03	-1.47E-02	4.16E-01	-8.23E-01	8.07E-01	9.84E-01	1.48E-01
ρ	s-block	4	1	1.95E-02	1.36E-03	1.20E-06	1.56E-04	1.05E-03	1.66E-03	4.10E-18	8.43E-01
ρ	transition	8	2	8.99E-01	3.77E-05	1.86E-08	6.93E-05	-9.81E-05	1.74E-04	5.86E-01	2.54E-01
ΔIP	s-block	4	1	1.73E+00	-1.35E-01	-1.66E-01	8.69E-02	-3.05E-01	3.57E-02	1.21E-01	5.46E-01
ΔIP	transition	8	2	1.65E+00	-4.16E-02	-1.89E-02	4.77E-02	-1.35E-01	5.19E-02	3.83E-01	1.22E-01
$\log(K_{OH})$	s-block	4	1	-1.02E+00	1.49E-01	1.24E-01	7.34E-02	4.64E-03	2.92E-01	4.31E-02	6.72E-01
$\log(K_{OH})$	transition	8	2	9.51E-01	3.64E-02	1.60E-02	4.15E-02	-4.50E-02	1.18E-01	3.81E-01	2.08E-02
MP	s-block	4	1	1.34E+00	-6.81E-04	-1.00E-06	3.80E-04	-1.43E-03	6.45E-05	7.34E-02	6.16E-01
MP	transition	8	2	1.23E+00	2.06E-05	1.96E-08	2.39E-04	-4.48E-04	4.89E-04	9.31E-01	8.84E-02
$pK_{sp}(CO_3)$	transition	7	2	-6.76E-01	-1.86E-01	-1.30E-01	8.77E-02	-3.58E-01	-1.42E-02	3.38E-02	2.73E-01
r	s-block	4	1	4.68E-01	5.35E-01	1.34E+00	3.88E-01	-2.26E-01	1.29E+00	1.68E-01	4.87E-01
r	transition	8	2	1.13E+00	1.40E-01	4.66E-01	6.95E-01	-1.22E+00	1.50E+00	8.40E-01	4.15E-01
σ_p	s-block	4	1	5.63E+00	-1.97E+01	-4.92E+02	3.71E+00	-2.69E+01	-1.24E+01	1.14E-07	5.97E-01
σ_p	transition	8	2	2.26E+00	-1.02E+01	-2.04E+02	3.01E+00	-1.61E+01	-4.31E+00	6.93E-04	6.76E-01
X_m	s-block	4	1	1.84E+00	-8.47E-01	-8.47E+00	5.19E-01	-1.87E+00	1.71E-01	1.03E-01	5.71E-01
X_m	transition	8	2	1.76E+00	-2.73E-01	-1.36E+00	6.32E-01	-1.51E+00	9.66E-01	6.66E-01	1.16E-03
X_m^2r	s-block	4	1	1.37E+00	-4.08E-01	-2.72E+00	6.99E-01	-1.78E+00	9.62E-01	5.59E-01	1.46E-01
X_m^2r	transition	8	2	7.40E-01	2.04E-01	1.29E-01	1.44E-01	-7.87E-02	4.88E-01	1.57E-01	2.88E-01
Z	s-block	4	1	1.40E+00	-3.29E-01	-3.29E-01	2.40E-01	-8.00E-01	1.41E-01	1.70E-01	4.85E-01
Z/AR	s-block	4	1	1.34E+00	-4.82E-01	-1.00E+00	2.70E-01	-1.01E+00	4.69E-02	7.41E-02	6.15E-01
Z/AR	transition	8	2	5.08E+00	-2.60E+00	-1.13E+01	5.72E-01	-3.72E+00	-1.47E+00	5.75E-06	6.53E-01
Z^2/r	s-block	4	1	1.15E+00	-7.73E-02	-2.82E-02	4.86E-02	-1.73E-01	1.80E-02	1.12E-01	5.58E-01
Z^2/r	transition	8	2	1.46E+00	-4.57E-02	-3.15E-02	8.46E-02	-2.11E-01	1.20E-01	5.89E-01	1.91E-01

[a]Number of metals included in model; [b]number of metals trimmed from the robust model based on total number for which data were available; [c]slope given per unit change in property; [d]standardized per interquartile range increase in property; SE: standard error; CL: 95% confidence limit.

Table S7. The associations between inference score relating CVD to metal exposures with the physical and chemical properties of metal ions using robust univariate regressions for stroke.

Property	Group	N[a]	N_{trim}[b]	Intercept	Slope[c]	Standardized Slope[d]	SE	Lower CL	Upper CL	P-Value	R^2
AN	s-block	4	1	1.24E+00	3.81E-02	1.23E-03	7.35E-03	2.37E-02	5.25E-02	2.18E-07	7.33E-01
AN	transition	8	2	2.51E+00	-1.04E-02	-5.00E-04	7.35E-03	-2.48E-02	3.95E-03	1.55E-01	1.60E-01
AN/ΔIP	s-block	4	1	1.51E+00	1.21E-01	1.94E-02	9.23E-02	-5.95E-02	3.02E-01	1.88E-01	4.37E-02
AN/ΔIP	transition	8	2	2.40E+00	-6.72E-02	-1.95E-02	5.16E-02	-1.68E-01	3.39E-02	1.93E-01	2.09E-01
AR	s-block	4	1	2.31E+00	-2.04E-01	-5.11E-01	3.32E-01	-8.54E-01	4.45E-01	5.37E-01	1.60E-01
AR	transition	8	2	4.73E+00	-1.96E+00	-9.80E+00	1.06E+00	-4.03E+00	1.13E-01	6.39E-02	2.85E-01
ΔE^0	s-block	4	1	4.16E+00	-8.55E-01	-5.03E+00	4.84E-01	-1.80E+00	9.35E-02	7.73E-02	1.85E-01
ΔE^0	transition	8	2	2.57E+00	-4.77E-01	-8.67E-01	3.78E-01	-1.22E+00	2.63E-01	2.07E-01	2.10E-01
ρ	s-block	4	1	1.51E+00	3.24E-04	2.88E-07	1.25E-04	7.83E-05	5.70E-04	9.77E-03	7.69E-01
ρ	transition	8	2	2.87E+00	-8.55E-05	-4.20E-08	8.77E-05	-2.57E-04	8.64E-05	3.30E-01	7.06E-02
ΔIP	s-block	4	1	1.46E+00	8.19E-02	1.01E-01	6.53E-02	-4.60E-02	2.10E-01	2.09E-01	4.41E-01
ΔIP	transition	8	2	1.84E+00	2.37E-02	1.08E-02	6.51E-02	-1.04E-01	1.51E-01	7.16E-01	1.38E-01
$\log(K_{OH})$	s-block	4	1	3.23E+00	-9.90E-02	-8.25E-02	4.54E-02	-1.88E-01	-9.89E-03	2.94E-02	7.03E-01
$\log(K_{OH})$	transition	8	2	2.06E+00	1.49E-03	6.54E-04	5.71E-02	-1.10E-01	1.13E-01	9.79E-01	7.25E-03
MP	s-block	4	1	1.67E+00	3.73E-04	5.74E-07	1.14E-04	1.49E-04	5.97E-04	1.09E-03	8.42E-01
MP	transition	8	2	1.68E+00	3.44E-04	3.27E-07	2.90E-04	-2.24E-04	9.12E-04	2.35E-01	2.29E-01
$pK_{sp}(CO_3)$	transition	7	2	-2.92E-02	-2.10E-01	-1.47E-01	1.10E-01	-4.27E-01	5.80E-03	5.65E-02	5.48E-01
r	s-block	4	1	2.21E+00	-2.84E-01	-7.11E-01	3.66E-01	-1.00E+00	4.32E-01	4.37E-01	2.32E-01
r	transition	8	2	2.86E+00	-9.10E-01	-3.03E+00	8.57E-01	-2.59E+00	7.71E-01	2.89E-01	9.57E-02
σ_p	s-block	4	1	3.73E+00	-9.63E+00	-2.41E+02	1.33E+00	-1.22E+01	-7.03E+00	4.11E-13	8.26E-01
σ_p	transition	7	2	2.19E+00	-1.62E+00	-3.24E+01	7.40E+00	-1.61E+01	1.29E+01	8.27E-01	8.46E-02
X_m	s-block	4	1	1.44E+00	4.75E-01	4.75E+00	4.19E-01	-3.45E-01	1.30E+00	2.56E-01	3.92E-01
X_m	transition	8	2	4.19E+00	-1.13E+00	-5.67E+00	7.39E-01	-2.58E+00	3.14E-01	1.25E-01	1.43E-01
X_m^2r	s-block	4	1	-4.86E-01	2.53E+00	1.69E+01	8.28E-02	2.37E+00	2.69E+00	4.51E-205	9.92E-01
X_m^2r	transition	8	2	2.66E+00	-1.86E-01	-1.18E-01	1.11E-01	-4.05E-01	3.21E-02	9.46E-02	3.22E-01
Z	s-block	4	1	1.53E+00	2.60E-01	2.60E-01	6.63E-02	1.30E-01	3.90E-01	8.74E-05	8.85E-01
Z/AR	s-block	4	1	1.64E+00	3.35E-01	6.98E-01	1.40E-01	6.02E-02	6.10E-01	1.69E-02	7.41E-01
Z/AR	transition	8	2	2.06E+00	9.38E-03	4.08E-02	5.86E-01	-1.14E+00	1.16E+00	9.87E-01	6.14E-02
Z^2/r	s-block	4	1	1.75E+00	5.94E-02	2.17E-02	2.29E-02	1.46E-02	1.04E-01	9.43E-03	7.71E-01
Z^2/r	transition	8	2	1.87E+00	4.47E-02	3.08E-02	1.12E-01	-1.75E-01	2.64E-01	6.90E-01	3.24E-02

[a]Number of metals included in model; [b]number of metals trimmed from the robust model based on total number for which data were available; [c]slope given per unit change in property; [d]standardized per interquartile range increase in property; SE: standard error; CL: 95% confidence limit.

Table S8. Comparisons of regression results from the various robust regression methods for cardiac arrhythmia.

			M estimation				MM estimation				S estimation				LTS			
**Property**	**Group**	**N**	**Slope**	**SE**	**P-Value**	**R^2**	**Slope**	**SE**	**P-Value**	**R^2**	**Slope**	**SE**	**P-Value**	**R^2**	**Slope**	**SE**	**P-Value**	**R^2**
AN	s-block	6	0.0059	0.005	0.2645	0.222	0.0059	0.0053	0.2645	0.2118	0.0059	0.005	0.2645	0.198	0.0059	0.005	0.2645	0.248
AN	transition	7	0.0065	0.002	0.0003	0.558	0.0065	0.0018	0.0003	0.5418	0.0065	0.002	0.0003	0.731	0.0059	9E-04	<.0001	0.835
AN/ΔIP	s-block	6	0.0225	0.022	0.3072	0.19	0.0225	0.022	0.3072	0.1788	0.0225	0.022	0.3072	0.159	0.0225	0.022	0.3072	0.166
AN/ΔIP	transition	7	0.0423	0.016	0.0075	0.461	0.0423	0.0158	0.0075	0.4528	0.0423	0.016	0.0075	0.541	0.0423	0.016	0.0075	0.608
AR	s-block	6	0.3469	0.309	0.2609	0.23	0.3469	0.3086	0.2609	0.2194	0.3469	0.309	0.2609	0.21	0.3469	0.309	0.2609	0.236
AR	transition	7	0.8748	0.347	0.0118	0.419	0.8748	0.3472	0.0118	0.4404	0.8748	0.347	0.0118	0.469	0.8748	0.347	0.0118	0.399
ΔE^0	s-block	6	-0.3654	1.093	0.7381	0.033	-0.3654	1.093	0.7381	0.036	-0.3654	1.093	0.7381	0.021	-0.3654	1.093	0.7381	0.402
ΔE^0	transition	7	0.0855	0.204	0.6746	0.002	0.0855	0.2036	0.6746	0.0101	0.0855	0.204	0.6746	0	-0.1091	0.138	0.429	0.135
ΔIP	s-block	6	-0.2192	0.164	0.1825	0.297	-0.2192	0.1644	0.1825	0.2912	-0.2192	0.164	0.1825	0.283	-0.2192	0.164	0.1825	0.431
ΔIP	transition	7	-0.0025	0.022	0.9085	9E-04	-0.0025	0.0221	0.9085	0	-0.0025	0.022	0.9085	0	0.0085	0.014	0.5432	0.085
ρ	s-block	6	0.0001	1E-04	0.2423	0.249	0.0001	0.0001	0.2423	0.2425	0.0001	1E-04	0.2423	0.241	0.0001	1E-04	0.2423	0.403
ρ	transition	7	0.0001	0	<.0001	0.682	0.0001	0	<.0001	0.5769	0.0001	0	<.0001	0.715	0.0001	0	<.0001	0.59
MP	s-block	6	-0.0001	4E-04	0.7543	0.029	-0.0001	0.0004	0.7543	0.0301	-0.0001	4E-04	0.7543	0.013	-0.0001	4E-04	0.7543	0.47
MP	transition	7	-0.0002	0	0.0002	0.227	-0.0001	0.0001	0.2317	0.2053	-0.0001	1E-04	0.2317	0.242	-0.0002	0	0.0002	0.57
X_m	s-block	6	-1.9266	1.29	0.1353	0.345	-1.9266	1.2899	0.1353	0.334	-1.9266	1.29	0.1353	0.341	-1.9266	1.29	0.1353	0.459
X_m	transition	7	0.5525	0.352	0.1167	0.281	0.5525	0.3522	0.1167	0.2344	0.5525	0.352	0.1167	0.153	0.5525	0.352	0.1167	0.037
X_m^2r	s-block	6	0.8951	0.888	0.3132	0.204	0.8951	0.8875	0.3132	0.2046	0.8951	0.888	0.3132	0.208	0.8951	0.888	0.3132	0.541
X_m^2r	transition	7	0.177	0.051	0.0005	0.622	0.177	0.0506	0.0005	0.5342	0.177	0.051	0.0005	0.641	0.177	0.051	0.0005	0.482
Z	s-block	6	-0.0082	0.264	0.9753	5E-04	-0.0082	0.2641	0.9753	0.0006	.	.	.	.	-0.0082	0.264	0.9753	0.357
Z^2/r	s-block	6	-0.0563	0.094	0.5507	0.108	-0.0563	0.0944	0.5507	0.0956	-0.0563	0.094	0.5507	0.093	-0.0563	0.094	0.5507	0.49
Z^2/r	transition	7	-0.0977	0.065	0.1328	0.21	-0.0977	0.065	0.1328	0.2297	-0.0977	0.065	0.1328	0.156	-0.0977	0.065	0.1328	0.026
Z/AR	s-block	6	-0.2793	0.485	0.5646	0.091	-0.2793	0.4848	0.5646	0.0888	-0.2793	0.485	0.5646	0.086	-0.7078	0.342	0.0386	0.573
Z/AR	transition	7	-0.3303	0.079	<.0001	0.259	-0.7054	0.3069	0.0215	0.4036	-0.7054	0.307	0.0215	0.425	-0.7054	0.307	0.0215	0.371
$\log(K_{OH})$	s-block	6	0.147	0.153	0.3366	0.201	0.147	0.153	0.3366	0.1976	0.147	0.153	0.3366	0.207	0.147	0.153	0.3366	0.599
$\log(K_{OH})$	transition	7	-0.0236	0.017	0.1663	0.239	-0.0236	0.0171	0.1663	0.193	-0.0236	0.017	0.1663	0.114	-0.0236	0.017	0.1663	0.033
$pK_{sp}(CO_3)$	s-block	4	-0.0435	0.057	0.445	0.219	.	.	.	.	.	.	.	.	.	.	.	.
$pK_{sp}(CO_3)$	transition	6	0.0352	0.006	<.0001	0.411	-0.0056	0.0418	0.8935	0.001	-0.0056	0.042	0.8935	0	0.0304	0.023	0.192	0.362
r	s-block	6	0.436	0.331	0.1878	0.292	0.436	0.331	0.1878	0.2833	0.436	0.331	0.1878	0.271	0.436	0.331	0.1878	0.431
r	transition	7	0.7319	0.366	0.0453	0.273	0.7319	0.3656	0.0453	0.3494	0.7319	0.366	0.0453	0.311	0.7319	0.366	0.0453	0.114
σ_p	s-block	6	-1.9623	5.088	0.6997	0.04	-1.9623	5.0877	0.6997	0.0432	-1.9623	5.088	0.6997	0.036	-8.3749	3.25	0.01	0.689
σ_p	transition	6	-5.5388	1.762	0.0017	0.639	-5.5388	1.7617	0.0017	0.5857	-5.5388	1.762	0.0017	0.679	-5.5388	1.762	0.0017	0.699

"-" symbol denotes that the simulation did not converge for the given method-property combination.

Table S9. Comparisons of regression results from the various robust regression methods for myocardial infarction.

			M estimation				MM estimation				S estimation				LTS			
Property	Group	N	Slope	SE	P-Value	R^2	Slope	SE	P-Value	R^2	Slope	SE	P-Value	R^2	Slope	SE	P-Value	R^2
AN	s-block	5	0.0504	0.015	0.0006	0.358	0.039	0.0416	0.3487	0.2371	0.039	0.042	0.3487	0.253	0.0504	0.015	0.0006	0.855
AN	transition	8	-0.0003	0.004	0.926	0.002	-0.0003	0.0035	0.926	0.0023	-0.0003	0.004	0.926	0	-0.0003	0.004	0.926	0.103
AN/ΔIP	s-block	5	0.2347	0.054	<.0001	0.515	0.2012	0.1821	0.2692	0.2994	0.2012	0.182	0.2692	0.327	0.2347	0.054	<.0001	0.904
AN/ΔIP	transition	8	-0.0007	0.025	0.9763	3E-04	-0.0007	0.0247	0.9763	0.0003	-0.0007	0.025	0.9763	0	-0.0007	0.025	0.9763	0.12
AR	s-block	5	1.0143	0.392	0.0096	0.363	1.0809	0.8016	0.1775	0.3624	1.0809	0.802	0.1775	0.38	1.0143	0.392	0.0096	0.77
AR	transition	8	0.2231	0.588	0.7043	0.019	0.2231	0.588	0.7043	0.0161	0.2231	0.588	0.7043	0	0.2231	0.588	0.7043	0.113
ΔE^0	s-block	5	-0.4954	1.256	0.6932	0.032	-0.4954	1.2556	0.6932	0.0363	-0.4954	1.256	0.6932	0	-0.4954	1.256	0.6932	0.196
ΔE^0	transition	8	-0.2292	0.232	0.3231	0.128	-0.2292	0.2319	0.3231	0.1214	-0.2292	0.232	0.3231	0.092	-0.2292	0.232	0.3231	0.034
ΔIP	s-block	5	-0.162	0.041	<.0001	0.338	-0.1874	0.2686	0.4854	0.1443	-0.1874	0.269	0.4854	0.128	-0.1874	0.269	0.4854	0.441
ΔIP	transition	8	0.0095	0.028	0.7362	0.018	0.0095	0.0283	0.7362	0.0172	0.0095	0.028	0.7362	0	0.0095	0.028	0.7362	0.029
ρ	s-block	5	0.0001	6E-04	0.8964	1E-04	0.0001	0.0006	0.8964	0.0028	0.0001	6E-04	0.8964	0	-0.0008	4E-04	0.0304	0.689
ρ	transition	8	0	0	0.6505	0.048	0	0	0.6505	0.0516	0	0	0.6505	0.056	0	0	0.6505	0.227
MP	s-block	5	-0.0009	5E-04	0.0473	0.207	-0.0005	0.0009	0.5817	0.1014	-0.0005	9E-04	0.5817	0.084	-0.0009	5E-04	0.0473	0.649
MP	transition	8	0.0001	1E-04	0.4328	0.097	0.0001	0.0001	0.4328	0.0976	0.0001	1E-04	0.4328	0.095	0.0001	1E-04	0.4328	0.538
X_m	s-block	5	-1.3847	1.094	0.2055	0.174	-1.1776	1.6507	0.4756	0.1475	-1.1776	1.651	0.4756	0.13	-1.1776	1.651	0.4756	0.423
X_m	transition	8	0.0281	0.354	0.9369	7E-04	0.0281	0.3544	0.9369	0.0005	0.0281	0.354	0.9369	0	0.0281	0.354	0.9369	0.019
X_m^2r	s-block	5	0.3577	1.674	0.8308	0.006	0.3577	1.6743	0.8308	0.0113	0.3577	1.674	0.8308	0	-2.2777	1.072	0.0336	0.681
X_m^2r	transition	8	0.0111	0.057	0.8448	0.006	0.0111	0.0568	0.8448	0.0054	0.0111	0.057	0.8448	0	0.0111	0.057	0.8448	0.005
Z	s-block	5	-0.6636	0.256	0.0094	0.113	-0.2401	0.579	0.6784	0.0663	-0.2401	0.579	0.6784	0.048	-0.6636	0.256	0.0094	0.762
Z^2/r	s-block	5	-0.1033	8E-04	<.0001	0.581	-0.0851	0.1438	0.554	0.119	-0.0851	0.144	0.554	0.111	-0.1598	0.066	0.0153	0.736
Z^2/r	transition	8	-0.0083	0.085	0.9218	9E-04	-0.0083	0.0851	0.9218	0.0007	-0.0083	0.085	0.9218	0	-0.0083	0.085	0.9218	0.059
Z/AR	s-block	5	-0.5746	0.023	<.0001	0.575	-0.6095	0.8271	0.4612	0.1637	-0.6095	0.827	0.4612	0.159	-0.8862	0.417	0.0335	0.681
Z/AR	transition	8	0.0395	0.584	0.9461	0.004	0.0395	0.5841	0.9461	0.0052	0.0395	0.584	0.9461	0	0.0395	0.584	0.9461	0.329
$log(K_{OH})$	s-block	5	0.1739	0.013	<.0001	0.528	0.2167	0.2459	0.3783	0.2106	0.2167	0.246	0.3783	0.21	0.2684	0.126	0.0337	0.68
$log(K_{OH})$	transition	8	0.007	0.027	0.7968	0.011	0.007	0.027	0.7968	0.0113	0.007	0.027	0.7968	0	0.007	0.027	0.7968	0.259
$pK_{sp}(CO_3)$	s-block	3	-0.0969	0.009	<.0001	0.945	-	-	-	-	.	.	.	.	.	.	.	.
$pK_{sp}(CO_3)$	transition	7	-0.0703	0.06	0.2386	0.169	-0.0703	0.0596	0.2386	0.1676	-0.0703	0.06	0.2386	0.122	-0.0703	0.06	0.2386	0.051
r	s-block	5	1.1697	0.532	0.0277	0.348	1.3175	0.9509	0.1659	0.3688	1.3175	0.951	0.1659	0.382	1.3175	0.951	0.1659	0.708
r	transition	8	0.1196	0.462	0.7958	0.01	0.1196	0.462	0.7958	0.0089	0.1196	0.462	0.7958	0	0.1196	0.462	0.7958	0.085
σ_p	s-block	5	-2.098	9.631	0.8276	0.005	-2.098	9.6306	0.8276	0.0097	-2.098	9.631	0.8276	0	10.6878	7.334	0.145	0.496
σ_p	transition	7	5.0104	0.981	<.0001	0.36	1.2891	3.6868	0.7266	0.0549	1.2891	3.687	0.7266	0.068	1.2891	3.687	0.7266	0.426

"-" symbol denotes that the simulation did not converge for the given method-property combination.

Table S10. Comparisons of regression results from the various robust regression methods for myocardial ischemia.

			M estimation				MM estimation				S estimation				LTS			
Property	Group	N	Slope	SE	P-Value	R^2	Slope	SE	P-Value	R^2	Slope	SE	P-Value	R^2	Slope	SE	P-Value	R^2
AN	s-block	5	0.0584	0.015	0.0001	0.571	0.0504	0.0308	0.1015	0.4751	0.0504	0.031	0.1015	0.508	0.0584	0.015	0.0001	0.84
AN	transition	9	0.0007	0.002	0.7457	0.015	0.0007	0.002	0.7457	0.007	0.0007	0.002	0.7457	0	0.0007	0.002	0.7457	0.187
AN/ΔIP	s-block	5	0.2757	0.042	<.0001	0.687	0.2525	0.1269	0.0467	0.5663	0.2525	0.127	0.0467	0.612	0.2757	0.042	<.0001	0.941
AN/ΔIP	transition	9	0.0055	0.014	0.701	0.021	0.0055	0.0143	0.701	0.01	0.0055	0.014	0.701	0	0.0055	0.014	0.701	0.244
AR	s-block	5	1.2392	0.561	0.0272	0.6	1.2392	0.561	0.0272	0.6099	1.2392	0.561	0.0272	0.62	2.5855	0.519	<.0001	0.873
AR	transition	9	0.0404	0.335	0.9042	0.002	0.0404	0.3353	0.9042	0.001	0.0404	0.335	0.9042	0	0.0404	0.335	0.9042	0.084
ΔE^0	s-block	5	0.0598	1.152	0.9586	0.003	0.0598	1.152	0.9586	0.003	0.0598	1.152	0.9586	0	1.5602	0.697	0.0252	0.715
ΔE^0	transition	9	-0.119	0.141	0.3984	0.033	-0.119	0.1409	0.3984	0.033	-0.119	0.141	0.3984	0	-0.119	0.141	0.3984	0.015
ΔIP	s-block	5	-0.2487	0.216	0.2491	0.324	-0.2487	0.2158	0.2491	0.3243	-0.2487	0.216	0.2491	0.347	-0.2851	0.108	0.0085	0.776
ΔIP	transition	9	-0.0171	0.012	0.1518	0.095	-0.0128	0.0169	0.4489	0.0366	-0.0128	0.017	0.4489	0	-0.0128	0.017	0.4489	0.291
ρ	s-block	5	0	6E-04	0.9355	0.001	0	0.0006	0.9355	0.0011	0	6E-04	0.9355	0	-0.0008	4E-04	0.0814	0.603
ρ	transition	9	0	0	0.4059	0.046	0	0	0.4059	0.0376	0	0	0.4059	0	0	0	0.4059	0.248
MP	s-block	5	-0.0002	8E-04	0.7878	0.026	-0.0002	0.0008	0.7878	0.0273	-0.0002	8E-04	0.7878	0.004	-0.0002	8E-04	0.7878	0.272
MP	transition	9	0	1E-04	0.5437	0.02	0	0.0001	0.5437	0.0177	0	1E-04	0.5437	0	0	1E-04	0.5437	0.004
X_m	s-block	5	-1.8231	0.567	0.0013	0.476	-1.6444	1.285	0.2007	0.3716	-1.6444	1.285	0.2007	0.402	-1.8231	0.567	0.0013	0.838
X_m	transition	9	0.1999	0.188	0.2884	0.052	0.1999	0.1883	0.2884	0.0582	0.1999	0.188	0.2884	0	0.1999	0.188	0.2884	0.214
X_m^2r	s-block	5	-0.0503	1.51	0.9734	0.002	-0.0503	1.5095	0.9734	0.0014	-0.0503	1.51	0.9734	0	-2.5986	0.262	<.0001	0.98
X_m^2r	transition	9	0.0257	0.031	0.409	0.052	0.0257	0.0312	0.409	0.0401	0.0257	0.031	0.409	0	0.0257	0.031	0.409	0.336
Z	s-block	5	-0.1504	0.526	0.7748	0.033	-0.1504	0.5257	0.7748	0.0335	-0.1504	0.526	0.7748	0.016	-0.1504	0.526	0.7748	0.468
Z^2/r	s-block	5	-0.0852	0.127	0.502	0.153	-0.0852	0.1268	0.502	0.1521	-0.0852	0.127	0.502	0.16	-0.1492	0.068	0.0278	0.708
Z^2/r	transition	9	-0.0234	0.047	0.6165	0.028	-0.0234	0.0468	0.6165	0.0176	-0.0234	0.047	0.6165	0	-0.0234	0.047	0.6165	0.403
Z/AR	s-block	5	-0.5912	0.728	0.417	0.199	-0.5912	0.7284	0.417	0.1986	-0.5912	0.728	0.417	0.207	-0.5912	0.728	0.417	0.656
Z/AR	transition	9	0.129	0.337	0.7015	0.014	0.129	0.3365	0.7015	0.0086	0.129	0.337	0.7015	0	0.129	0.337	0.7015	0.009
$\log(K_{OH})$	s-block	5	0.2224	0.211	0.2918	0.299	0.2224	0.2109	0.2918	0.2891	0.2224	0.211	0.2918	0.308	0.2665	0.111	0.0164	0.742
$\log(K_{OH})$	transition	9	0.0145	0.014	0.3012	0.07	0.0145	0.014	0.3012	0.054	0.0145	0.014	0.3012	0	0.0145	0.014	0.3012	0.091
$pK_{sp}(CO_3)$	s-block	3	-0.0965	0.087	0.2647	0.539	-	-	-	-	.	.	.	.	.	.	.	.
$pK_{sp}(CO_3)$	transition	8	0.007	0.034	0.8372	0.001	0.0472	0.0746	0.5274	0.0229	0.007	0.034	0.8372	0	-0.0612	0.024	0.011	0.618
r	s-block	5	1.4671	0.686	0.0324	0.587	1.4671	0.6858	0.0324	0.5946	1.4671	0.686	0.0324	0.596	3.2952	0.514	<.0001	0.921
r	transition	9	0.1252	0.258	0.6276	0.03	0.1252	0.2581	0.6276	0.0164	0.1252	0.258	0.6276	0	0.1252	0.258	0.6276	0.384
σ_p	s-block	5	-0.9505	8.67	0.9127	0.003	-0.9505	8.6696	0.9127	0.0024	-0.9505	8.67	0.9127	0	10.3909	6.933	0.1339	0.529
σ_p	transition	8	1.7191	1.8	0.3396	0.095	1.7191	1.8002	0.3396	0.0442	1.7191	1.8	0.3396	0	1.7191	1.8	0.3396	0.37

“-“ symbol denotes that the simulation did not converge for the given method-property combination.

Table S11. Comparisons of regression results from the various robust regression methods for stroke.

			M estimation				MM estimation				S estimation				LTS			
Property	Group	N	Slope	SE	P-Value	R^2	Slope	SE	P-Value	R^2	Slope	SE	P-Value	R^2	Slope	SE	P-Value	R^2
AN	s-block	5	0.0362	0.014	0.0118	0.567	0.0362	0.0144	0.0118	0.5917	0.0362	0.014	0.0118	0.66	0.0381	0.007	<.0001	0.734
AN	transition	10	-0.0104	0.007	0.1552	0.197	-0.0104	0.0073	0.1552	0.184	-0.0104	0.007	0.1552	0.196	-0.0104	0.007	0.1552	0.16
AN/ΔIP	s-block	5	0.1214	0.092	0.1885	0.324	0.1214	0.0923	0.1885	0.3166	0.1214	0.092	0.1885	0.286	0.1214	0.092	0.1885	0.044
AN/ΔIP	transition	10	-0.0672	0.052	0.1927	0.183	-0.0672	0.0516	0.1927	0.1751	-0.0672	0.052	0.1927	0.204	-0.0672	0.052	0.1927	0.209
AR	s-block	5	0.3086	0.515	0.549	0.086	0.3086	0.5151	0.549	0.0818	0.3086	0.515	0.549	0	-0.2045	0.332	0.5374	0.16
AR	transition	10	-1.9602	1.058	0.0639	0.279	-1.9602	1.0578	0.0639	0.2474	-1.9602	1.058	0.0639	0.25	-1.9602	1.058	0.0639	0.285
ΔE^0	s-block	5	-0.8545	0.484	0.0773	0.472	-0.8545	0.4837	0.0773	0.4601	-0.8545	0.484	0.0773	0.435	-0.8545	0.484	0.0773	0.185
ΔE^0	transition	10	0.4499	0.523	0.3897	0.072	0.4499	0.5231	0.3897	0.0731	0.4499	0.523	0.3897	0.04	-0.4768	0.378	0.2067	0.21
ΔIP	s-block	5	0.0819	0.065	0.2094	0.186	0.1065	0.1427	0.4556	0.1518	0.1065	0.143	0.4556	0.117	0.0819	0.065	0.2094	0.441
ΔIP	transition	10	0.0416	0.043	0.3271	0.049	0.0237	0.0651	0.7159	0.0227	0.0237	0.065	0.7159	0	0.0237	0.065	0.7159	0.138
ρ	s-block	5	0.0005	2E-04	0.0007	0.673	0.0005	0.0002	0.0007	0.725	0.0005	2E-04	0.0007	0.771	0.0003	1E-04	0.0098	0.77
ρ	transition	10	-0.0001	1E-04	0.3296	0.086	-0.0001	0.0001	0.3296	0.085	-0.0001	1E-04	0.3296	0.058	-0.0001	1E-04	0.3296	0.071
MP	s-block	5	0.0004	1E-04	0.0011	0.503	0.0006	0.0004	0.1273	0.4016	0.0006	4E-04	0.1273	0.422	0.0004	1E-04	0.0011	0.842
MP	transition	10	0.0003	3E-04	0.2347	0.124	0.0003	0.0003	0.2347	0.1175	0.0003	3E-04	0.2347	0.1	0.0003	3E-04	0.2347	0.229
X_m	s-block	5	0.4754	0.419	0.2562	0.167	0.5985	0.8933	0.5029	0.1283	0.5985	0.893	0.5029	0.088	0.4754	0.419	0.2562	0.392
X_m	transition	10	-1.1341	0.739	0.1247	0.221	-1.1341	0.7386	0.1247	0.1915	-1.1341	0.739	0.1247	0.198	-1.1341	0.739	0.1247	0.143
X_m^2r	s-block	5	1.3589	0.451	0.0026	0.712	1.3589	0.4512	0.0026	0.7216	1.3589	0.451	0.0026	0.715	2.5308	0.083	<.0001	0.992
X_m^2r	transition	10	-0.1863	0.111	0.0946	0.245	-0.1863	0.1114	0.0946	0.2353	-0.1863	0.111	0.0946	0.266	-0.1863	0.111	0.0946	0.322
Z	s-block	5	0.2601	0.066	<.0001	0.521	0.4177	0.2095	0.0461	0.515	0.4177	0.21	0.0461	0.549	0.2601	0.066	<.0001	0.885
Z	transition	10	-0.0466	0.365	0.8985	0.017	-0.272	0.5656	0.6306	0.027	-0.272	0.566	0.6306	0	-0.272	0.566	0.6306	0.003
Z^2/r	s-block	5	0.0594	0.023	0.0094	0.461	0.0923	0.0618	0.1352	0.3893	0.0923	0.062	0.1352	0.401	0.0594	0.023	0.0094	0.771
Z^2/r	transition	10	0.0447	0.112	0.6901	0.017	0.0447	0.1121	0.6901	0.0117	0.0447	0.112	0.6901	0	0.0447	0.112	0.6901	0.032
Z/AR	s-block	5	0.3351	0.14	0.0169	0.456	0.4743	0.397	0.2322	0.3033	0.4743	0.397	0.2322	0.306	0.3351	0.14	0.0169	0.741
Z/AR	transition	10	0.2273	0.363	0.5309	0	0.0094	0.586	0.9872	0.0002	0.0094	0.586	0.9872	0	0.0094	0.586	0.9872	0.061
$\log(K_{OH})$	s-block	5	-0.1274	0.129	0.3212	0.243	-0.1274	0.1285	0.3212	0.24	-0.1274	0.129	0.3212	0.233	-0.099	0.045	0.0294	0.703
$\log(K_{OH})$	transition	10	-0.0042	0.05	0.9331	0.002	0.0015	0.0571	0.9792	0	0.0015	0.057	0.9792	0	0.0015	0.057	0.9792	0.007
$pK_{sp}(CO_3)$	s-block	3	-0.114	0.013	<.0001	0.947	-	-	-	-	.	.	.	.	.	.	.	.
$pK_{sp}(CO_3)$	transition	9	-0.2105	0.11	0.0565	0.169	-0.029	0.1334	0.8277	0.0183	-0.029	0.133	0.8277	0.024	-0.2105	0.11	0.0565	0.548
r	s-block	5	0.2701	0.634	0.6703	0.043	0.2701	0.6344	0.6703	0.0376	0.2701	0.634	0.6703	0	-0.2842	0.366	0.4369	0.232
r	transition	10	-0.9095	0.858	0.2888	0.116	-0.9095	0.8575	0.2888	0.1091	-0.9095	0.858	0.2888	0.096	-0.9095	0.858	0.2888	0.096
σ_p	s-block	5	-7.6664	2.744	0.0052	0.543	-7.6664	2.7438	0.0052	0.6351	-7.6664	2.744	0.0052	0.699	-9.635	1.329	<.0001	0.826
σ_p	transition	9	-6.8566	6.008	0.2537	0.029	-1.6205	7.4023	0.8267	0.0113	-1.6205	7.402	0.8267	0	-1.6205	7.402	0.8267	0.085

"-" symbol denotes that the simulation did not converge for the given method-property combination.

Table S12. Comparisons of regression results from the various robust regression methods for thrombosis.

			M estimation				MM estimation				S estimation				LTS			
**Property**	**Group**	**N**	**Slope**	**SE**	**P-Value**	**R^2**	**Slope**	**SE**	**P-Value**	**R^2**	**Slope**	**SE**	**P-Value**	**R^2**	**Slope**	**SE**	**P-Value**	**R^2**
AN	s-block	5	0.0533	0.037	0.1481	0.387	0.0533	0.0368	0.1481	0.3524	0.0533	0.037	0.1481	0.28	0.0136	0.024	0.5658	0.142
AN	transition	10	-0.0006	0.006	0.9181	1E-04	-0.0006	0.0063	0.9181	0.001	-0.0006	0.006	0.9181	0	-0.0006	0.006	0.9181	0.33
AN/ΔIP	s-block	5	0.2012	0.186	0.2785	0.224	0.2012	0.1857	0.2785	0.2364	0.2012	0.186	0.2785	0.163	0.0766	0.089	0.3872	0.272
AN/ΔIP	transition	10	-0.0004	0.043	0.9924	0.002	-0.0004	0.0432	0.9924	0.0043	-0.0004	0.043	0.9924	0	-0.0004	0.043	0.9924	0.423
AR	s-block	5	0.4295	0.34	0.2058	0.263	0.6533	0.9587	0.4956	0.123	0.6533	0.959	0.4956	0.049	0.4295	0.34	0.2058	0.445
AR	transition	10	-0.2463	0.958	0.7971	0.001	-0.2463	0.9582	0.7971	0.0001	-0.2463	0.958	0.7971	0	2.2079	0.544	<.0001	0.662
ΔE^0	s-block	5	0.2067	1.301	0.8737	0.014	0.2067	1.3005	0.8737	0.0086	0.2067	1.301	0.8737	0	0.2254	0.549	0.6815	0.078
ΔE^0	transition	10	-0.0081	0.416	0.9845	2E-04	-0.0081	0.416	0.9845	0.0008	-0.0081	0.416	0.9845	0	-0.0081	0.416	0.9845	0.148
ΔIP	s-block	5	-0.1347	0.087	0.1213	0.238	-0.0971	0.2883	0.7362	0.0464	-0.0971	0.288	0.7362	0	-0.1347	0.087	0.1213	0.546
ΔIP	transition	10	-0.0416	0.048	0.3831	0.097	-0.0416	0.0477	0.3831	0.0967	-0.0416	0.048	0.3831	0.096	-0.0416	0.048	0.3831	0.122
ρ	s-block	5	-0.0002	3E-04	0.5436	0.02	0.0004	0.0006	0.5663	0.0844	0.0004	6E-04	0.5663	0	0.0014	2E-04	<.0001	0.843
ρ	transition	10	0	1E-04	0.403	0.095	0	0.0001	0.5862	0.0635	0	1E-04	0.5862	0.076	0	1E-04	0.5862	0.254
MP	s-block	5	0.0008	8E-04	0.302	0.15	0.0008	0.0008	0.302	0.2368	0.0008	8E-04	0.302	0.122	-0.0007	4E-04	0.0734	0.616
MP	transition	10	0	2E-04	0.9312	2E-04	0	0.0002	0.9312	0.0019	0	2E-04	0.9312	0	0	2E-04	0.9312	0.088
X_m	s-block	5	-0.8471	0.519	0.1029	0.239	-0.9183	1.731	0.5958	0.0899	-0.9183	1.731	0.5958	0.017	-0.8471	0.519	0.1029	0.571
X_m	transition	10	-0.2727	0.632	0.6662	0.019	-0.2727	0.6321	0.6662	0.0154	-0.2727	0.632	0.6662	0	-0.2727	0.632	0.6662	0.001
X_m^2r	s-block	5	-0.0163	1.711	0.9924	0.006	-0.0163	1.7108	0.9924	0.0009	-0.0163	1.711	0.9924	0	-0.4083	0.699	0.5591	0.146
X_m^2r	transition	10	-0.038	0.098	0.697	0.013	-0.038	0.0976	0.697	0.009	-0.038	0.098	0.697	0	0.2045	0.145	0.157	0.288
Z	s-block	5	0.3923	0.56	0.4834	0	0.3923	0.5597	0.4834	0.1144	0.3923	0.56	0.4834	0	-0.3294	0.24	0.1698	0.485
Z	transition	10	-0.2048	0.431	0.6343	0.034	-0.2048	0.4305	0.6343	0.0396	-0.2048	0.431	0.6343	0.246	-0.2048	0.431	0.6343	0.248
Z^2/r	s-block	5	0.0302	0.153	0.8435	8E-04	0.0302	0.1532	0.8435	0.0032	0.0302	0.153	0.8435	0	-0.0773	0.049	0.1121	0.558
Z^2/r	transition	10	-0.0457	0.085	0.5894	0.062	-0.0457	0.0846	0.5894	0.0604	-0.0457	0.085	0.5894	0.067	-0.0457	0.085	0.5894	0.191
Z/AR	s-block	5	0.175	0.906	0.8468	0.002	0.175	0.9059	0.8468	0.0027	0.175	0.906	0.8468	0	-0.4815	0.27	0.0741	0.615
Z/AR	transition	10	-0.2306	0.438	0.5988	0.064	-0.2306	0.4384	0.5988	0.0462	-0.2306	0.438	0.5988	0.132	-2.5961	0.572	<.0001	0.653
$\log(K_{OH})$	s-block	5	0.1485	0.073	0.0431	0.275	0.003	0.2798	0.9914	0.004	0.003	0.28	0.9914	0	0.1485	0.073	0.0431	0.672
$\log(K_{OH})$	transition	10	0.0364	0.042	0.3807	0.082	0.0364	0.0415	0.3807	0.0739	0.0364	0.042	0.3807	0.055	0.0364	0.042	0.3807	0.021
$pK_{sp}(CO_3)$	s-block	3	-0.1205	0.181	0.5065	0.298	-	-	-	-	.	.	.	.	.	.	.	.
$pK_{sp}(CO_3)$	transition	9	-0.0631	0.097	0.5152	0.045	-0.0631	0.097	0.5152	0.0472	-0.0631	0.097	0.5152	0	-0.1861	0.088	0.0338	0.273
r	s-block	5	0.5346	0.388	0.1682	0.316	0.6495	1.1766	0.581	0.0914	0.6495	1.177	0.581	0.014	0.5346	0.388	0.1682	0.487
r	transition	10	0.1399	0.695	0.8404	0.022	0.1399	0.695	0.8404	0.022	0.1399	0.695	0.8404	0.021	0.1399	0.695	0.8404	0.415
σ_p	s-block	5	2.2646	4.531	0.6172	0.02	-5.2262	9.3696	0.577	0.0805	-5.2262	9.37	0.577	0	-19.6662	3.708	<.0001	0.598
σ_p	transition	9	-10.2186	3.012	0.0007	0.246	-5.6593	5.2736	0.2832	0.2899	-10.2186	3.012	0.0007	0.447	-10.2186	3.012	0.0007	0.676

“-“ symbol denotes that the simulation did not converge for the given method-property combination.

Table S13. Average results from 1000 Monte Carlo simulations of the LTS regression. Percent error denotes the relative error between the LTS slope from the data model fit compared with the LTS slope from the Monte Carlo simulation for cardiac arrhythmia.

Property	Group	Slope	SE[a]	Simulated Slope Variance[b]	Percent Error
AN	s-block	5.82E-03	4.64E-03	4.27E-05	6.57E-01
AN	transition	5.81E-03	1.18E-03	3.99E-06	8.23E-01
AN/ΔIP	s-block	2.33E-02	2.01E-02	7.07E-04	-3.75E+00
AN/ΔIP	transition	4.40E-02	1.60E-02	5.02E-04	-4.07E+00
AR	s-block	3.55E-01	2.74E-01	1.62E-01	-2.20E+00
AR	transition	8.89E-01	3.63E-01	2.31E-01	-1.64E+00
ΔE^0	s-block	-3.61E-01	1.09E+00	2.61E+00	1.34E+00
ΔE^0	transition	-1.03E-01	1.46E-01	3.58E-02	5.88E+00
ρ	s-block	1.38E-04	1.05E-04	3.11E-08	-8.23E+00
P	transition	6.25E-05	1.77E-05	1.12E-09	3.14E-01
ΔIP	s-block	-2.19E-01	1.79E-01	7.56E-02	-1.70E-02
ΔIP	transition	8.63E-03	1.74E-02	6.04E-04	-1.49E+00
$\log(K_{OH})$	s-block	1.51E-01	1.41E-01	4.02E-02	-2.42E+00
$\log(K_{OH})$	transition	-2.49E-02	1.82E-02	5.46E-04	-5.35E+00
MP	s-block	-1.37E-04	3.86E-04	2.87E-07	-1.76E+01
MP	transition	-1.80E-04	5.54E-05	5.04E-09	4.61E-01
$pK_{sp}(CO_3)$	transition	3.11E-02	2.68E-02	1.91E-03	-2.59E+00
r	s-block	4.69E-01	3.69E-01	2.91E-01	-7.47E+00
r	transition	7.18E-01	3.98E-01	2.53E-01	1.89E+00
σ_p	s-block	-8.40E+00	3.13E+00	2.17E+01	-2.43E-01
σ_p	transition	-5.49E+00	1.61E+00	5.66E+00	8.13E-01
X_m	s-block	-1.99E+00	1.14E+00	2.72E+00	-3.13E+00
X_m	transition	5.21E-01	3.75E-01	2.51E-01	5.78E+00
$X_m^2 r$	s-block	8.95E-01	8.46E-01	1.90E+00	-4.52E-02
$X_m^2 r$	transition	1.80E-01	5.20E-02	7.05E-03	-1.46E+00
Z	s-block	-1.29E-02	2.91E-01	1.47E-01	-5.74E+01
Z/AR	s-block	-6.89E-01	3.65E-01	3.11E-01	2.61E+00
Z/AR	transition	-7.02E-01	3.19E-01	1.77E-01	5.11E-01
Z^2/r	s-block	-5.22E-02	9.49E-02	1.79E-02	7.27E+00
Z^2/r	transition	-9.05E-02	6.88E-02	9.61E-03	7.34E+00

[a]Square root of the mean of the sum of the squares of SE for each fo the 1000 simulations; [b]Variance of the 1000 simulated slopes

Table S14. Average results from 1000 Monte Carlo simulations of the LTS regression. Percent error denotes the relative error between the LTS slope from the data model fit compared with the LTS slope from the Monte Carlo simulation for myocardial infarction.

Property	Group	Slope	SE[a]	Simulated Slope Variance[b]	Percent Error
AN	s-block	5.03E-02	1.61E-02	9.28E-04	1.29E-01
AN	transition	-6.00E-04	3.66E-03	3.50E-05	-8.30E+01
AN/ΔIP	s-block	2.36E-01	6.01E-02	1.24E-02	-7.14E-01
AN/ΔIP	transition	-1.54E-03	2.53E-02	1.69E-03	-1.10E+02
AR	s-block	1.03E+00	4.61E-01	6.74E-01	-1.97E+00
AR	transition	1.86E-01	5.61E-01	7.00E-01	1.68E+01
ΔE^0	s-block	-4.31E-01	1.24E+00	5.70E+00	1.30E+01
ΔE^0	transition	-2.16E-01	2.54E-01	1.34E-01	5.94E+00
ρ	s-block	-8.55E-04	3.27E-04	3.13E-07	-1.10E+00
P	transition	-1.49E-05	4.34E-05	6.99E-09	1.59E+01
ΔIP	s-block	-2.04E-01	2.40E-01	2.15E-01	-8.73E+00
ΔIP	transition	1.24E-02	3.59E-02	7.23E-03	-3.03E+01
$\log(K_{OH})$	s-block	2.73E-01	1.40E-01	6.72E-02	-1.88E+00
$\log(K_{OH})$	transition	5.19E-03	3.00E-02	2.78E-03	2.54E+01
MP	s-block	-9.26E-04	4.99E-04	7.45E-07	-2.20E+00
MP	transition	1.00E-04	9.81E-05	2.05E-08	4.99E+00
$pK_{sp}(CO_3)$	transition	-7.41E-02	5.93E-02	5.92E-03	-5.45E+00
r	s-block	1.38E+00	8.24E-01	2.53E+00	-4.46E+00
r	transition	1.36E-01	4.52E-01	5.18E-01	-1.37E+01
σ_p	s-block	1.11E+01	6.41E+00	1.21E+02	-3.74E+00
σ_p	transition	1.11E+00	3.77E+00	2.60E+01	1.40E+01
X_m	s-block	-1.20E+00	1.54E+00	9.93E+00	-2.00E+00
X_m	transition	2.09E-02	3.55E-01	3.41E-01	2.54E+01
X_m^2r	s-block	-2.29E+00	9.26E-01	2.78E+00	-4.94E-01
X_m^2r	transition	1.50E-02	6.85E-02	2.10E-02	-3.53E+01
Z	s-block	-6.49E-01	2.70E-01	1.52E-01	2.14E+00
Z/AR	s-block	-8.89E-01	4.71E-01	6.68E-01	-3.44E-01
Z/AR	transition	-2.27E-02	5.69E-01	7.78E-01	1.58E+02
Z^2/r	s-block	-1.60E-01	7.43E-02	1.77E-02	-6.00E-02
Z^2/r	transition	-8.48E-03	8.21E-02	1.57E-02	-1.55E+00

[a]Square root of the mean of the sum of the squares of SE for each fo the 1000 simulations; [b]Variance of the 1000 simulated slopes

Table S15. Average results from 1000 Monte Carlo simulations of the LTS regression. Percent error denotes the relative error between the LTS slope from the data model fit compared with the LTS slope from the Monte Carlo simulation for myocardial ischemia.

Property	Group	Slope	SE[a]	Simulated Slope Variance[b]	Percent Error
AN	s-block	5.91E-02	1.70E-02	8.95E-04	-1.13E+00
AN	transition	5.55E-04	2.48E-03	1.84E-05	1.54E+01
AN/ΔIP	s-block	2.76E-01	4.56E-02	7.16E-03	-1.15E-01
AN/ΔIP	transition	7.08E-03	1.70E-02	6.57E-04	-2.89E+01
AR	s-block	2.56E+00	3.34E-01	3.57E-01	1.11E+00
AR	transition	1.12E-02	3.95E-01	2.98E-01	7.23E+01
ΔE^0	s-block	1.57E+00	6.09E-01	1.52E+00	-5.74E-01
ΔE^0	transition	-1.12E-01	1.61E-01	4.72E-02	6.00E+00
ρ	s-block	-7.41E-04	3.55E-04	4.13E-07	1.52E+00
P	transition	1.87E-05	2.71E-05	1.93E-09	-3.41E+00
ΔIP	s-block	-2.88E-01	1.30E-01	6.67E-02	-1.16E+00
ΔIP	transition	-1.20E-02	2.04E-02	1.27E-03	6.04E+00
$\log(K_{OH})$	s-block	2.69E-01	1.27E-01	5.09E-02	-8.39E-01
$\log(K_{OH})$	transition	1.37E-02	1.73E-02	7.40E-04	5.76E+00
MP	s-block	-2.91E-04	6.91E-04	1.26E-06	-3.32E+01
MP	transition	4.15E-05	8.95E-05	1.42E-08	8.40E+00
$pK_{sp}(CO_3)$	transition	-6.28E-02	2.46E-02	1.34E-03	-2.54E+00
r	s-block	3.30E+00	3.22E-01	3.43E-01	-9.39E-02
r	transition	1.17E-01	3.19E-01	2.02E-01	6.27E+00
σ_p	s-block	9.85E+00	5.94E+00	1.02E+02	5.23E+00
σ_p	transition	1.61E+00	2.15E+00	1.43E+01	6.21E+00
X_m	s-block	-1.77E+00	7.22E-01	2.05E+00	2.81E+00
X_m	transition	1.99E-01	2.27E-01	1.20E-01	5.61E-01
X_m^2r	s-block	-2.59E+00	2.15E-01	1.84E-01	3.08E-01
X_m^2r	transition	2.60E-02	4.38E-02	6.36E-03	-1.08E+00
Z	s-block	-1.60E-01	4.87E-01	5.18E-01	-6.63E+00
Z/AR	s-block	-5.51E-01	6.29E-01	1.23E+00	6.83E+00
Z/AR	transition	1.47E-01	3.92E-01	3.29E-01	-1.41E+01
Z^2/r	s-block	-1.48E-01	7.66E-02	1.71E-02	7.16E-01
Z^2/r	transition	-2.37E-02	5.69E-02	5.47E-03	-1.02E+00

[a]Square root of the mean of the sum of the squares of SE for each fo the 1000 simulations; [b]Variance of the 1000 simulated slopes

Table S16. Average results from 1000 Monte Carlo simulations of the LTS regression. Percent error denotes the relative error between the LTS slope from the data model fit compared with the LTS slope from the Monte Carlo simulation for stroke.

Property	Group	Slope	SE[a]	Simulated Slope Variance[b]	Percent Error
AN	s-block	3.85E-02	8.44E-03	2.33E-04	-1.18E+00
AN	transition	-1.10E-02	7.54E-03	1.14E-04	-5.57E+00
AN/ΔIP	s-block	1.21E-01	8.06E-02	1.93E-02	1.57E-01
AN/ΔIP	transition	-6.93E-02	4.85E-02	4.32E-03	-3.06E+00
AR	s-block	-1.98E-01	3.13E-01	3.22E-01	3.08E+00
AR	transition	-2.06E+00	1.09E+00	1.84E+00	-5.23E+00
ΔE^0	s-block	-8.86E-01	5.18E-01	9.48E-01	-3.74E+00
ΔE^0	transition	-4.56E-01	4.43E-01	2.88E-01	4.33E+00
ρ	s-block	3.31E-04	1.03E-04	3.21E-08	-2.15E+00
P	transition	-8.35E-05	9.04E-05	1.64E-08	2.32E+00
ΔIP	s-block	7.56E-02	7.73E-02	2.47E-02	7.80E+00
ΔIP	transition	2.16E-02	7.15E-02	1.45E-02	8.72E+00
$\log(K_{OH})$	s-block	-9.89E-02	5.24E-02	8.75E-03	6.53E-02
$\log(K_{OH})$	transition	2.59E-03	5.79E-02	6.97E-03	-7.42E+01
MP	s-block	3.82E-04	1.25E-04	4.91E-08	-2.41E+00
MP	transition	3.52E-04	2.86E-04	1.24E-07	-2.06E+00
$pK_{sp}(CO_3)$	transition	-2.20E-01	1.12E-01	2.42E-02	-4.66E+00
r	s-block	-2.91E-01	3.66E-01	4.84E-01	-2.24E+00
r	transition	-9.07E-01	8.43E-01	1.26E+00	2.84E-01
σ_p	s-block	-9.59E+00	1.34E+00	5.20E+00	4.74E-01
σ_p	transition	-1.37E+00	7.51E+00	1.02E+02	1.56E+01
X_m	s-block	4.55E-01	5.17E-01	1.20E+00	4.21E+00
X_m	transition	-1.12E+00	7.13E-01	1.02E+00	1.14E+00
$X_m^2 r$	s-block	2.53E+00	5.38E-02	1.06E-02	2.06E-01
$X_m^2 r$	transition	-1.88E-01	1.09E-01	2.62E-02	-7.66E-01
Z	s-block	2.59E-01	7.03E-02	9.85E-03	3.26E-01
Z	transition	-2.50E-01	5.77E-01	3.80E-01	7.91E+00
Z/AR	s-block	3.38E-01	1.58E-01	7.50E-02	-8.80E-01
Z/AR	transition	-1.02E-02	7.12E-01	1.33E+00	2.09E+02
Z^2/r	s-block	6.10E-02	2.56E-02	1.81E-03	-2.66E+00
Z^2/r	transition	4.56E-02	1.16E-01	3.06E-02	-2.12E+00

[a]Square root of the mean of the sum of the squares of SE for each fo the 1000 simulations; [b]Variance of the 1000 simulated slopes

Table S17. Average results from 1000 Monte Carlo simulations of the LTS regression. Percent error denotes the relative error between the LTS slope from the data model fit compared with the LTS slope from the Monte Carlo simulation for thrombosis.

Property	Group	Slope	SE[a]	Simulated Slope Variance[b]	Percent Error
AN	s-block	1.35E-02	2.26E-02	1.53E-03	9.22E-01
AN	transition	-6.38E-04	5.48E-03	6.45E-05	7.49E-01
AN/ΔIP	s-block	7.37E-02	9.07E-02	2.69E-02	3.80E+00
AN/ΔIP	transition	2.00E-03	3.64E-02	2.27E-03	5.82E+02
AR	s-block	4.39E-01	3.92E-01	4.71E-01	-2.23E+00
AR	transition	2.24E+00	5.66E-01	4.82E-01	-1.61E+00
ΔE^0	s-block	2.24E-01	6.42E-01	1.47E+00	7.89E-01
ΔE^0	transition	3.02E-03	4.25E-01	2.89E-01	1.37E+02
ρ	s-block	1.35E-03	1.32E-04	5.03E-08	1.90E-01
P	transition	3.54E-05	7.23E-05	1.07E-08	6.08E+00
ΔIP	s-block	-1.41E-01	1.05E-01	4.73E-02	-4.31E+00
ΔIP	transition	-3.92E-02	4.71E-02	5.09E-03	5.82E+00
$\log(K_{OH})$	s-block	1.51E-01	8.09E-02	2.25E-02	-1.52E+00
$\log(K_{OH})$	transition	3.50E-02	4.28E-02	3.43E-03	3.81E+00
MP	s-block	-7.05E-04	2.92E-04	2.34E-07	-3.50E+00
MP	transition	2.02E-05	2.42E-04	9.16E-08	2.05E+00
$pK_{sp}(CO_3)$	transition	-1.84E-01	1.08E-01	2.49E-02	9.19E-01
r	s-block	5.07E-01	4.54E-01	6.34E-01	5.24E+00
r	transition	1.65E-01	5.69E-01	5.41E-01	-1.78E+01
σ_p	s-block	-1.98E+01	2.98E+00	2.97E+01	-6.95E-01
σ_p	transition	-1.05E+01	3.39E+00	2.13E+01	-2.75E+00
X_m	s-block	-8.56E-01	6.63E-01	2.04E+00	-1.02E+00
X_m	transition	-3.02E-01	6.46E-01	9.21E-01	-1.06E+01
X_m^2r	s-block	-4.22E-01	8.16E-01	2.41E+00	-3.38E+00
X_m^2r	transition	2.04E-01	9.91E-02	2.46E-02	1.06E-01
Z	s-block	-3.19E-01	2.17E-01	9.16E-02	3.12E+00
Z	transition	-1.99E-01	4.41E-01	2.39E-01	2.73E+00
Z/AR	s-block	-4.99E-01	2.87E-01	2.54E-01	-3.61E+00
Z/AR	transition	-2.60E+00	2.95E-01	2.48E-01	-7.69E-02
Z^2/r	s-block	-7.69E-02	5.19E-02	8.80E-03	4.67E-01
Z^2/r	transition	-4.39E-02	8.93E-02	1.78E-02	3.89E+00

[a]Square root of the mean of the sum of the squares of SE for each fo the 1000 simulations; [b]Variance of the 1000 simulated slopes

References

The Comparative Toxicogenomics Database. 2013. http://ctdbase.org. Accessed 21 February, 2013.

Dean JA (ed). 1999. Lange's Handbook of Chemistry (15th Edition), McGraw-Hill. Section 3; Table 3.2 Physical Constants of Inorganic Compounds.

James AM, Lord, MP. 1992. Macmillan's Chemical and Physical Data, Macmillan, London, UK.

Kaye GWC, Laby TH. 1993. Tables of physical and chemical constants, Longman, London, UK, 15th edition.

Lide DR (ed). 2003. CRC Handbook of Chemistry and Physics, 84th Edition. CRC Press. Boca Raton, Florida. Section 4, Properties of the Elements and Inorganic Compounds; Melting, boiling, and critical temperatures of the elements.

Mendes LF, Bastos EL, Stevani CV. 2010. Prediction of metal cation toxicity to the bioluminescent fungus gerronema viridilucens. Environmental Toxicology and Chemistry 29:2177-2181.

Wolterbeek HT, Verburg TG. 2001. Predicting metal toxicity revisited: general properties vs. specific effects. The Science of the Total Environment 279:87-115.
